# Engineering Robust, Porous Guar Gum Hydrogels by One-Step Mild Synthesis: Impact of Porogen Choice on Rheology and Sustained Gastroretentive Amoxicillin Delivery

**DOI:** 10.3390/gels11100785

**Published:** 2025-10-01

**Authors:** Fátima Díaz-Carrasco, M.-Violante De-Paz, Matea Katavić, Estefanía García-Pulido, Álvaro Santos-Medina, Lucía Muíña-Ramil, M.-Gracia García-Martín, Elena Benito

**Affiliations:** Departamento de Química Orgánica y Farmacéutica, Universidad de Sevilla, C/Prof. García González, n.º 2, 41012 Sevilla, Spain; fdiaz4@us.es (F.D.-C.); graciagm@us.es (M.-G.G.-M.); ebenito@us.es (E.B.)

**Keywords:** guar gum hydrogel, interpenetrating polymer network (IPN), Diels–Alder crosslinking, one-pot synthesis, porosity modulation, PEG porogen, sucrose porogen, gastroretentive drug delivery systems, GRDDS

## Abstract

This study introduces a single-step method to synthesize guar gum-based interpenetrating polymer network (IPN) hydrogels, achieving simultaneous Diels–Alder crosslinking and amoxicillin (AMOX) encapsulation under mild conditions. To evaluate the influence of porogen addition on IPN structure, drug loading and release, twenty-one formulations were developed, including AMOX loading (25% or 40% *w*/*w* relative to the polymer) and biocompatible porogens incorporation [polyethylene glycol (PEG) or sucrose at 5%, 10%, or 50% *w*/*w*]. All crosslinked IPN hydrogels formed robust gels, unlike non-crosslinked controls. Porogen choice strongly influenced hydrogel performance: PEG quadrupled the swelling index while enhancing storage modulus (up to 10,054 Pa) and complex viscosity (up to 1302 Pa·s), whereas high sucrose concentrations produced soft, ductile networks with critical strains above 20% and swelling indices up to 1895%. All hydrogels released AMOX at levels above MIC_50_ for *H. pylori*. PEG-based IPN provided superior drug delivery profiles, with extended AMOX release (t_50_ up to 15.5 h at pH 5.0), while sucrose-rich matrices exhibited faster burst release and disintegration. Single-step (pre-loading) AMOX during synthesis improved release control compared to post-loading. These findings highlight the potential of one-pot IPN synthesis with porogen modulation offering a promising gastroretentive platforms for sustained AMOX delivery against *H. pylori*.

## 1. Introduction

Cancer is a leading global health burden, with stomach cancer being among the most prevalent forms of the disease [1].

Chronic infection with *Helicobacter pylori* (*H. pylori*) is the main established risk factor for gastric cancer [2]. Research published in the relevant literature demonstrates that the eradication of *H. pylori* infection results in a reduction in the incidence of stomach cancer [3]. In this regard, recommended first-line treatment, known as “triple therapy”, includes two antibiotics (AMOX and clarithromycin) in conjunction with a proton pump inhibitor [4]. In the last decade, this treatment has shown a significant decrease in effectiveness due to factors such as non-compliance, inadequate dosing and, especially, antibiotic resistance of *H. pylori* [5].

Out of all the antibiotics, amoxicillin (AMOX) seems to have the lowest resistance rate, which makes it a perfect candidate to strengthen this therapy [6]. Nonetheless, to be effective, AMOX needs to maintain consistent active pharmaceutical levels above the minimal inhibitory concentration (MIC = 0.023 mg/mL) [7] for extended periods. To achieve this, frequent dosing of the drug is necessary, a requirement that leads to non-adherence treatment for many patients, often resulting in suboptimal eradication rates [4].

Considering the aforementioned points, new pharmacological strategies are necessary to fight against *H. pylori* infection without inducing microbial resistance and improving prescription compliance. Gastro-retentive drug delivery systems (GRDDS) are increasing in relevance, enabling sustained and localized release on the site of action [8]. This results in a reduction in the dose frequency. Furthermore, GRDDS can be administrated in an oral pharmaceutical form, which is the most convenient for patients, and it is also known for its low cost and safe administration [9].

GRDDS employ a range of mechanisms to extend gastric residence time, including swelling, floating, density, or mucoadhesion, among others. The utilization of superporous hydrogels and mucoadhesive polymers has emerged as a promising avenue for the development of these strategies. These materials possess the capacity to rapidly swell, buoy, and establish strong adhesive interactions with the gastric mucosa [6].

The utilization of mucoadhesive GRDDS in conjunction with buoyancy techniques is a common practice for the purpose of achieving gastric retention. It is widely accepted that the desired floating behavior of the dosage form can be achieved through the addition of effervescent combinations or swelling enhancers. Nevertheless, this final approach is not without its challenges. It is imperative to ensure that consistent swelling behavior is exhibited under varying gastric conditions, as well as achieving complete drug release [10]. Another possibility is the incorporation of effervescent combinations. Nevertheless, all formulations proposed for the manufacture of floating AMOX-loaded GRDDS (e.g., bilayer tablets, capsular devices, and raft systems) incorporate at least two or three additional excipients to guarantee such buoyancy during the expected release time [6,8].

Another option available on the market is the combination of floating and swelling systems [11]. However, the available data on the direct impact of the swellable GRDDS on drug bioavailability remains limited [12]. Moreover, it is evident that the cost-effectiveness of these systems is questionable, and their development is arduous [11].

Previous mucoadhesive AMOX-loaded GRDDS have been prepared with the incorporation of one or various bioadhesive polymers, including, but not limited to, chitosan (CTS), pectin, Carbopol^®^, polyacrylic acid (PAA), and natural gums. These systems have been prepared by different methods such as ionic gelation [13,14], emulsion solvent evaporation [15,16], or spray drying [17]. It is important to note that the majority of these systems demonstrate low drug loading (DL) capacities [6]. This is attributable to the limited space available for drug incorporation, which limits their capacity to deliver adequate amounts of therapeutic agents per unit dosage [18]. In addition, the employment of sophisticated techniques and meticulous excipient selection is imperative, resulting in escalated production costs and an augmented level of intricacy [10].

As demonstrated in previous research, porosity is a necessary property for sustained-release systems when controlled release of active pharmaceutical ingredients (API) at high doses is required, as in the case of AMOX [19]. In fact, it has been shown that porosity ensures both high encapsulation capacity and high swelling capacity [20,21,22]. The incorporation of porogens into IPN is a critical strategy for engineering porous matrices, being instrumental in determining the three-dimensional structure of the network—specifically pore size, porosity, density, and mechanical properties [23]—and consequently influences drug release kinetics. These porogen agents are hydrophilic materials that may include, for example, sucrose (Suc), lactose, dextrin, cellulose, sodium chloride or polyethylene glycol (PEG) [24]. Upon contact with water, these materials undergo immediate dissolution, resulting in their self-elimination and the subsequent formation of porous structures that serve as the medium for the encapsulation of the drugs. The porosity of the hydrogel is contingent upon the dimensions of the porogens employed in its fabrication [25,26]. In addition, the system hydrophilicity is increased when porogens are used [27].

In the present work, we have investigated the development of novel biocompatible materials that offer buoyancy, swelling, and bioadhesion capacity for AMOX sustained-release GRDDS to improve bioavailability and overall pharmacokinetics and to achieve the successful eradication of *H. pylori* in infected patients. The core systems proposed have been obtained as semi-interpenetrating polymer networks (IPNs), which are constituted by a water-soluble biopolymer [Polymer 1, guar gum (GG) [28]] that is intertwined with another polymer (Polymer 2). GG gels via its galactopyranose residues, which bind to water molecules leading to a colloidal solution. Moreover, GG lacks toxicity, has mucoadhesive properties and is highly biodegradable, making it an ideal choice for biological purposes [19,29]. The objective of this study is to enhance the drug release efficiency and swelling rate of systems prepared in a single step by combining novel cross-linked polymer networks and porogen agents to afford superporous, stable, and strong microstructures. PEG and sucrose were selected as porogen agents for their specific properties. PEG has strong water solubility, established role in forming well-defined pore walls [30] and also enhance the solubility of hydrophobic drugs, thereby improving their bioavailability and pharmacokinetics [31,32]. Low-molecular-weight PEG acts as a cosolvent due to its high miscibility with water [33]. Sucrose has the ability to generate open, interconnected pores upon dissolution in water [34], thereby facilitating water ingress and promoting the release of the encapsulated AMOX.

The preparation of IPN requires the synthesis of Polymer 2 via a click Diels–Alder (DA) reaction [35] from a difurfuryl monomer and a dimaleimide monomer. The process of crosslinking between a trifurfuryl crosslinking agent and the dimaleimide monomer results in the formation of a three-dimensional structure within the hydrogel, characterized by highly porous polymeric networks. This facilitates rapid swelling, allowing retention in the stomach, and resisting gastric emptying over extended periods [36].

It is significant that selection of the starting products employed in the formation of the novel IPN, as well as the synthetic methodology, will support the circular economy by enabling recovery, recycling, and reintegration into production cycles. GG is a low-cost product entirely derived from renewable plant biomass [37], and monomers with renewable carbon backbone [38,39,40] will be used for the synthesis of Polymer 2. Furthermore, the DA reaction used to synthesize Polymer 2 is still one of the most important green synthetic methodologies due to its theoretical 100% atom economy [41]. This reaction is known for its thermal reversibility, chemoselectivity, and high efficiency [42], making it particularly suitable for the formation of covalent bonds in crosslinked hydrogels. It can proceed at room temperature and in aqueous media, eliminating the need for potentially toxic solvents [43], and it does not generate side products. Remarkably, it can also undergo retro-DA reactions, and hence degradation, at temperatures as low as 50 °C [44].

In summary, this approach seeks to overcome the current shortage of GRDDS formulations for AMOX by proposing single-step preparation of delivery systems that involve highly efficient and eco-friendly reactions and ensure effective drug release to address *H. pylori* infection.

## 2. Results and Discussion

### 2.1. Synthesis of IPN Hydrogels

**Figure 1:**
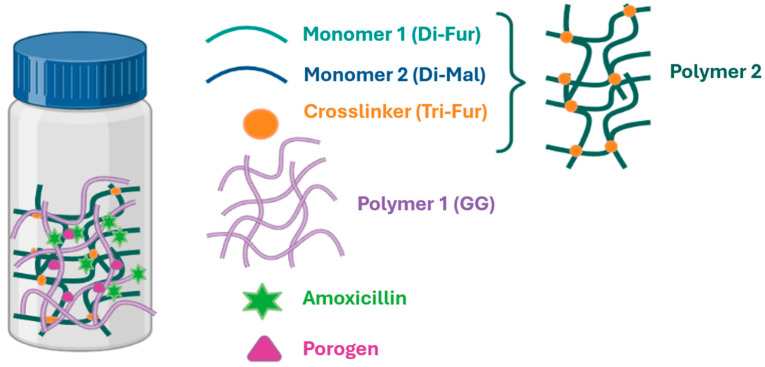
Schematic representation of single-step preparation of porous, drug-loaded IPN systems.

To prepare the various IPNs (Figure 1), Polymer 2 was synthesized from the monomers Di-Fur [45] and Di-Mal [46], together with the crosslinker Tri-Fur [22] via a DA reaction conducted in a single step within a colloidal solution of GG (Polymer 1) in distilled water. It is important to note that these single-step, or one-pot, reactions offer several advantages that are especially attractive for biomedical applications. As highlighted by Dhand et al. [47], one-pot reactions streamline synthesis and reduce purification requirements, further enhancing their suitability for biomedical hydrogel preparation.

As outlined in Section 1, the presence of a porogenic agent is instrumental in determining the 3D structure of the network [23] and consequently influences drug release kinetics. In this comparative study, we systematically evaluated the impact of both nature (PEG and sucrose) and concentration (5%, 10%, and 50% *w*/*w* relative to the polymer matrix) of porogens on key properties such as porosity, rheological behavior, disintegration profile, and drug release kinetics. The porogens were incorporated during IPN synthesis, with PEG added in its liquid form and sucrose introduced as an aqueous solution (0.5 g mL^−1^), ensuring homogeneous distribution within the matrix.

**Figure 2:**
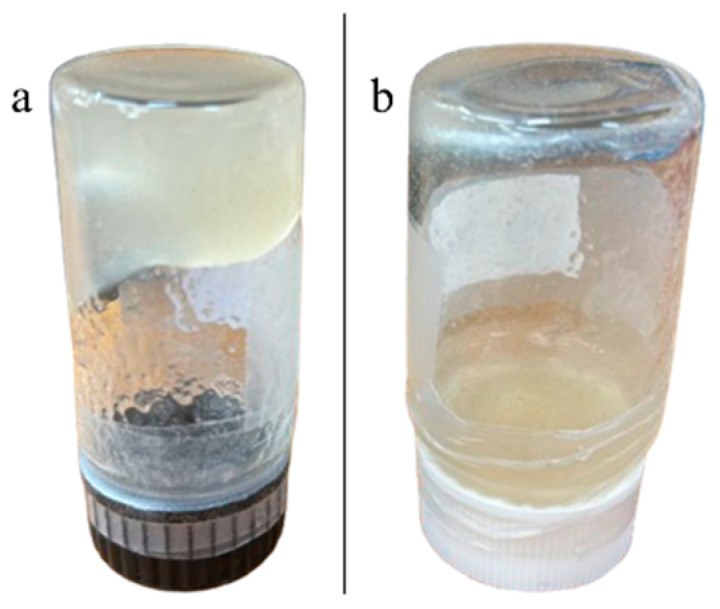
Appearance of the hydrogels after 7 days of preparation: (**a**) gel-like structure of IPN **B3.4** (**GG-DA-PEG_5_**) and (**b**) liquid state of control system **GG-PEG_5_**.

For the preparation of AMOX-loaded IPN, two drug loading levels were evaluated: 25% and 40% (*w*/*w* relative to the polymer matrix). IPN were synthesized both with and without porogens, and AMOX was incorporated either by co-loading during IPN formation or by post-loading into preformed networks. In the co-loading approach, AMOX was dissolved in dimethyl sulfoxide (DMSO) at a concentration of 0.5 g·mL^−1^ and added directly to the reaction mixture, taking advantage of the orthogonality of the reaction chemistry to combine drug encapsulation and matrix synthesis in a single step. Alternatively, post-loading was performed by introducing AMOX into preformed IPN, allowing for the comparative assessment of both loading strategies. The presence of a porous medium, achieved through the use of porogens, was found to be essential for effective AMOX encapsulation [48]. This methodology is particularly relevant for pharmaceutical applications, as it enables the development of drug delivery systems with tunable porosity, mechanical integrity, and controlled release profiles. All formulations, detailed in Section 4 (see also Appendix A), exhibited a stable gel-like consistency that was maintained over time (Figure 2a). For comparative purposes, control systems lacking Polymer 2 were also prepared; although these initially exhibited a gel-like consistency, they transitioned to a completely liquid state within one week, as shown for **GG-PEG_5_** in Figure 2b, underscoring the critical role of Polymer 2 in maintaining the structural stability and rheological properties of the IPN matrix.

### 2.2. Rheological Analysis: Influence of Porogens and AMOX Loading in IPN Hydrogels

To systematically evaluate the main rheological trends in the developed hydrogels, a comparative analysis was performed across three groups: simple GG systems (with and without porogens, Table 1), non-drug-loaded IPN systems (blanks, Table 2), and AMOX-loaded IPN (Table 3). This design allows for direct comparison of the effects of porogen type, porogen concentration, and AMOX content on the mechanical and viscoelastic properties of the hydrogels, as detailed in Table 1, Table 2 and Table 3. The rheological analysis of the hydrogels demonstrated how the network structure, the presence and type of porogen (PEG or sucrose), and the amount of AMOX together determine the mechanical and physical properties of the IPN systems.

For simple GG systems (Table 1), the addition of porogens such as PEG or sucrose generally increases critical strain (${\dot{\gamma }}_{c})$, storage modulus at 2π rad∙s ($G_{1}^{\prime })$, loss modulus at 2π rad∙s $\left(G_{1}^{\prime \prime }\right)$, complex viscosity at 2π rad∙s ($\eta_{1}^{*})$, and plateau modulus ($G_{N}^{0})$ compared to pure GG. For instance, **GG-PEG_50_** exhibits the highest $G_{1}^{\prime }$ (1545 Pa) and $G_{N}^{0}$ (1990 Pa) among the simple GG formulations, indicating that higher PEG content results in more robust gels. Similarly, sucrose at high concentration (**GG-Suc_50_**) yields the highest ${\dot{\gamma }}_{c}$ (12.91%), suggesting enhanced flexibility and resistance to deformation.

A comparison between simple GG hydrogels and non-drug-loaded IPN blanks (Table 2) reveals clear effects of both network crosslinking and porogen incorporation on rheological properties. The 3D network of Polymer 2 is a structural scaffold that limits the mobility of guar gum chains, which results in an increase in the system capability to withstand higher tensions without experiencing deformity [43]. When a porogen was not used, forming an IPN by crosslinking DA within GG significantly increases the stiffness and resistance of the hydrogel. Thus, $G_{1}^{\prime }$ rises from 489 Pa in simple GG to 1227 Pa in the GG-DA IPN (**B1.2**), and $\eta_{1}^{*}$ increases from 79.3 Pa·s to 208.9 Pa·s. This is consistent with literature reports that crosslinking enhances mechanical strength and structural integrity in hydrogel systems [49].

**Figure 3:**
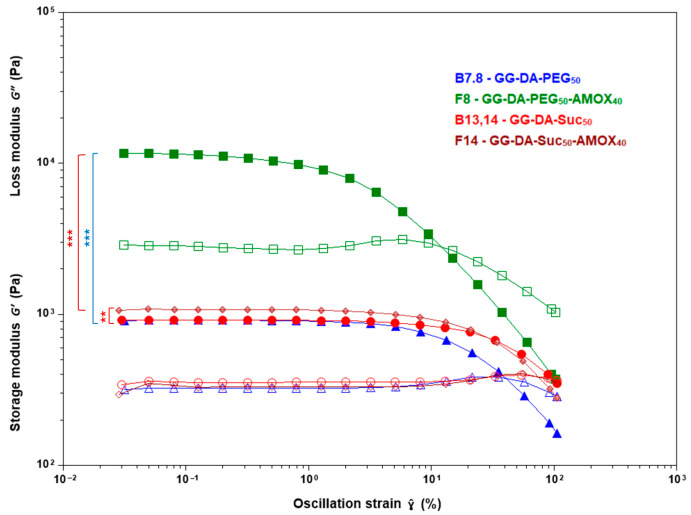
Effect of porogen type (PEG or sucrose) on critical strain during strain sweep tests in hydrogel systems. Asterisks on the figure indicate significant differences between each IPN; **: *p* < 0.01, and ***: *p* < 0.001.

The incorporation of porogens such as PEG or Suc into IPN hydrogels significantly influences their rheological properties (Table 2, Figure 3). Upon the addition of PEG at 5%, 10%, and 50% concentrations, there is a marked increase in ${\dot{\gamma }}_{c}$ to approximately 5.1% for all PEG-containing samples, indicating a substantial improvement in the hydrogel’s flexibility and ability to withstand deformation before yielding. $G_{1}^{\prime }$ decreases with increasing PEG content, from 1214 Pa at 5% PEG to 959 Pa at 50% PEG, reflecting a progressive softening of the network. Sucrose, when used as a porogen at the same concentrations (5%, 10%, and 50%), produces an even more pronounced effect on ${\dot{\gamma }}_{c}$. The critical strain increases from 8.13% at 5% Suc to 20.60% at 50% Suc, showing that sucrose dramatically enhances the hydrogel’s ductility and tolerance to deformation. $G_{1}^{\prime }$ for sucrose-containing samples ranges from 925 Pa at 5% Suc to 994 Pa at 50% Suc, indicating a moderate reduction compared to the non-porogen sample, but less pronounced than with PEG. This is consistent with reports that sucrose creates a more open, interconnected pore structure, enhancing flexibility but reducing stiffness [50]. Both porogens decrease the elastic and viscous moduli, complex viscosity, and plateau modulus, indicating a softer and more flexible hydrogel network.

The impact of AMOX loading on the rheological properties of the IPN hydrogels is strongly influenced by both the type of porogen and the drug concentration (Table 3).

Across all IPN systems, whether loaded with AMOX or not, the loss tangent [tan (δ)] values remain below 0.4, indicating predominantly elastic, solid-like behavior [42]. Notably, sucrose-based systems tend to exhibit slightly higher tan (δ) values compared to their PEG counterparts, reflecting a more viscoelastic and less purely elastic character. For example, in non-drug-loaded IPN blanks, tan (δ) ranges from 0.35 to 0.39 for PEG systems (**B3.4**, **B5.6**, **B7.8**) and from 0.36 to 0.38 for sucrose systems (**B9.10**, **B11.12**, **B13.14**). In AMOX-loaded formulations, the highest tan (δ) observed is 0.40 for **F9** (GG-DA-Suc_5_-AMOX_25_), while most PEG-based systems maintain tan (δ) values between 0.23 and 0.34.

**Figure 4:**
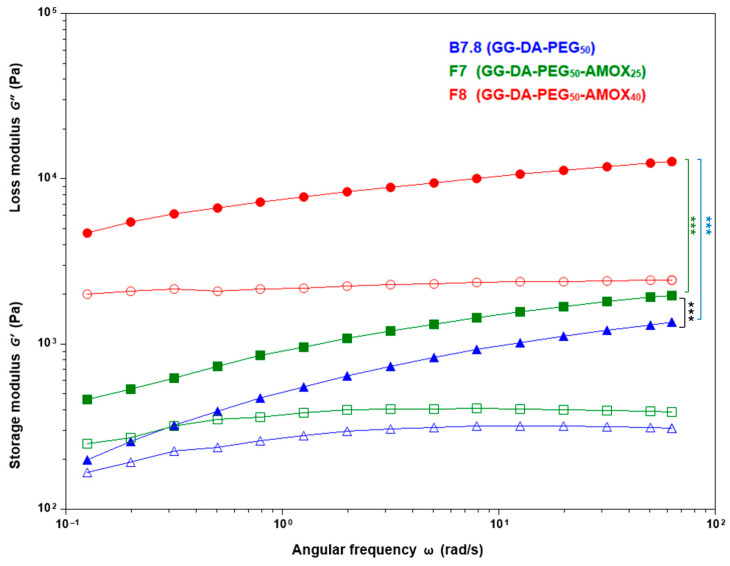
Influence of AMOX concentration on storage modulus (filled color symbols) and loss modulus (empty color symbols) during frequency sweep tests in hydrogels systems incorporating PEG as porogen at 5% and 50% concentrations. Asterisks on the figure indicate significant differences between each IPN; ***: *p* < 0.001.

In PEG-based IPN, increasing the AMOX content from 25% to 40% leads to a marked rise in both $G^{\prime }$ and $G_{N}^{0}$. This effect is exemplified by formulations such as **F4** (PEG 5%, AMOX 40%) which exhibits a $G_{1}^{\prime }$ of 4657 Pa and $G_{N}^{0}$ of 6112 Pa, and **F8** (PEG 50%, AMOX 40%) which achieves the highest values recorded, with $G_{1}^{\prime }$ at 10,054 Pa and $G_{N}^{0}$ at 12,702 Pa. Additionally, $\eta_{1}^{*}$ in **F8** reaches 1302.3 Pa·s, a significant increase compared to the blank GG-DA-PEG_50_ system (**B7.8**), which exhibits a $G_{1}^{\prime }$ of 959 Pa and a $\eta_{1}^{*}$ of 123.4 Pa·s. The rheological data for PEG-containing systems **F4**, **F6**, and **F8** demonstrate a threshold effect, where the combination of high PEG and AMOX content synergistically enhances the hydrogel network (Table 3, Figure 4). The marked increase in stiffness and mechanical strength of PEG–AMOX formulations compared to Suc–AMOX counterparts is due to multiple reinforcing mechanisms. PEG chains interpenetrate and entangle with GG and the crosslinked DA network, enhancing network cohesion. In addition, as previously mentioned, PEG promotes AMOX solubilization, enabling the drug to act as both a physical filler and an interfacial adhesive through extensive hydrogen bonding and ion–dipole interactions with PEG, GG, and the oligo(ethylene glycol) domains of the DA network. PEG’s dual role as a structural reinforcer and mediator of drug–polymer interactions leads to a synergistic strengthening of the hydrogel matrix, in contrast to sucrose-based systems that lack such reinforcement. Similar synergistic effects of drug and porogen on network mechanics have been reported in other drug-loaded hydrogel systems [51].

In contrast, for sucrose-based IPN, increasing the AMOX content has a less pronounced effect on $G^{\prime }$ and $G_{N}^{0}$, but consistently increases ${\dot{\gamma }}_{c}$, suggesting that higher drug loading enhances the flexibility of the hydrogel rather than its stiffness. For instance, **F12** (Suc_10_, AMOX_40_) and **F14** (Suc_50_, AMOX_40_) show ${\dot{\gamma }}_{c}$ values of 20.95% and 21.12%, respectively, among the highest in the study. These findings demonstrate that AMOX loading can be used as an effective parameter to modulate the mechanical properties of IPN hydrogels, with the specific effects depending on the type and concentration of porogen present.

The overall findings from the rheological studies are summarized in Table 4. Intertwining GG with Polymer 2 network is essential for maintaining the structural integrity and mechanical strength of the hydrogels. The type and concentration of porogen enable precise tuning of the rheological properties.

### 2.3. Swelling Studies on IPN Matrices

Swelling capacity is a key attribute for gastro-retentive drug delivery systems (GRDDS), as the resulting increase in volume can impede premature passage through the pylorus and promote prolonged gastric retention. Additionally, if the swollen system maintains a density below 1.004 g/mL, it remains buoyant in simulated gastric fluid (SGF), further prolonging its gastric residence [8].

The analysis of the swelling index (SI) data for lyophilized IPN hydrogels shows that the incorporation of porogens such as PEG or sucrose significantly increases the SI compared to the base **GG-DA** hydrogel (497%, Table 5). For example, the addition of PEG at 5% (**GG-DA-PEG_5_**) results in a SI of 1585% with a very low error (0.5%), indicating both high swelling and reproducibility. The SI peaks at 2002% for **GG-DA-PEG_10_**, exhibiting a moderate error margin (7.3%), while at the highest PEG concentration (**GG-DA-PEG_50_**), the SI remains elevated. The addition of sucrose as a porogen also enhances the SI, with **GG-DA-Suc_10_** achieving 2058% with a low error of 1.1%. These results demonstrate that both the type and concentration of porogen are critical in determining the swelling behavior and reproducibility of the hydrogels.

One study reported a direct correlation between increased porosity and enhanced swelling rate upon the incorporation of porogens into hydrogel formulations [52]. PEG acts as a pore-forming agent, increasing the porosity and hydrophilicity of hydrogels, which in turn enhances their swelling capacity [53]. Sucrose has also been employed as a pore-forming agent, facilitating the development of macropores within hydrogel structures [54]. Studies have shown that the swelling behavior of PEG hydrogels is closely related to the network structure and the amount of PEG incorporated, with higher PEG content generally leading to greater water uptake and larger mesh sizes [55]. However, excessive porogen content can introduce heterogeneity and slightly reduce swelling capacity as reflected at the highest PEG and sucrose concentrations.

To conclude, the results confirm that the swelling behavior of IPN hydrogels can be finely tuned by adjusting the type and concentration of porogen. Intermediate concentrations of PEG or sucrose (e.g., PEG_10_ or Suc_10_) provide the most favorable combination of high SI (2002% and 2058%, respectively) and reproducibility (errors of 7.3% and 1.1%, respectively). These observations are consistent with the literature, which emphasizes the importance of network structure and porosity in optimizing hydrogel swelling properties for biomedical and drug delivery applications [26].

### 2.4. Floatability Studies

Floatability tests were performed on all AMOX-loaded GG-DA IPN formulations (**F1–F14**) during drug release studies at 37 °C, 100 rpm, at both pH 1.2 and pH 5.0. All formulations exhibited an immediate floating lag time (FLT = 0 min), indicating instant buoyancy upon introduction to the medium, except for the sucrose-based **IPN F11** (GG-DA-Suc_10_-AMOX_25_) at pH 5.0, which showed a delayed FLT of 3 min.

In terms of total floating time, both GG-DA-AMOX formulations (**F1**: AMOX_25_ and **F2**: AMOX_40_) remained buoyant for more than 8 h at both pH. Following this period, the hydrogels began to disintegrate; however, small fragments continued to float up to 24 h.

When PEG was incorporated as a porogen (**F3–F8**), all PEG-based formulations remained buoyant for 24 h at pH 5.0 before significant disintegration was observed. In contrast, at pH 1.2, all PEG-containing IPNs (**F3–F8**) floated for 8 h; however, the formulations with the highest PEG content—**F7** (GG-DA-PEG_50_-AMOX_25_) and **F8** (GG-DA-PEG_50_-AMOX_40_)—began to fragment after this period.

In the sucrose-based formulations (**F9–F14**), at pH 5.0, all sucrose-based IPNs except **F14** (Suc_50_-AMOX_40_, which sank at 3 h) remained buoyant for 24 h. Notably, **F10** (Suc_5-_AMOX_40_) persisted as a single, intact floating piece throughout the entire incubation period. In contrast, reduced buoyancy was observed at pH 1.2. Specifically, **F14** (Suc_50-_AMOX_40_) sank after 1 h, and **F11** (Suc_10_-AMOX_25_) after 2 h. By 24 h, most sucrose-containing IPNs had disintegrated, leaving only small fragments (<25% of the original mass).

In summary, all formulations (**F1–F14**) demonstrated immediate buoyancy (FLT = 0 min) upon introduction to the medium, with the exception of GG-DA-Suc_10_-AMOX_25_ (**F11**) at pH 5.0, which exhibited a floating lag time of approximately 3 min. Both the GG-DA-AMOX (**F1**, **F2**) and PEG-based IPNs (**F3–F8**) maintained robust buoyancy, particularly under neutral conditions (pH 5.0), where they remained afloat for extended periods. In contrast, sucrose-based IPNs (**F9–F14**) displayed reduced floating stability at acidic pH (pH 1.2), with several formulations sinking or disintegrating within a few hours. However, at pH 5.0, most sucrose-based systems retained their buoyancy for up to 24 h, except for GG-DA-Suc_50_-AMOX_40_ (**F14**), which lost buoyancy after 3 h.

### 2.5. Degradation Kinetics of IPN Formulations Under Simulated Physiological Conditions

The degradability of IPN formulations is essential for human safety, ensuring that residual materials do not accumulate or cause adverse effects post-therapy. In GRDDS for *H. pylori* treatment, a balance must be achieved: the IPN should remain stable for 12–24 h at pH 5.0—reflecting the elevated gastric pH during infection [6]—while still being degradable to prevent long-term retention. Thus, controlled degradability directly supports both therapeutic efficacy and patient safety.

Under simulated physiological conditions (37 °C, 150 rpm), the degradation kinetics of the IPN formulations demonstrated clear dependencies on both the presence and type of porogen, as well as the environmental pH. As an initial result, all systems exhibited slight degradation at 3 h post-incubation and fully disintegrated after 24 h in nearly all cases (p. 4, [56]). Formulations without porogen (**F1** and **F2**) showed the fastest degradation, with 50% mass loss occurring within 3.94 to 6.16 h. Specifically, **F2** (GG-DA-AMOX_40_) at pH 1.2 was the least stable, degrading to half its mass in just 3.94 h, while **F1** (GG-DA-AMOX_25_) at pH 5.0 degraded in 6.16 h. The greater degradability of **F1** and **F2** is attributed to their denser, less porous network structure, which increases brittleness and susceptibility to cracking. In contrast, formulations with PEG or sucrose as porogens exhibit higher porosity and flexibility, resulting in enhanced mechanical stability and slower degradation [57]. These rapid degradation rates indicate that non-porogen formulations prepared using a one-pot strategy are unsuitable for applications requiring extended gastric retention.

The incorporation of PEG as a porogen significantly enhanced the stability of the IPN matrices (p. 4, [56]). At pH 5.0, PEG-based formulations such as **F3** (PEG_5_-AMOX_25_) and **F4** (PEG_5_-AMOX_40_) exhibited the slowest degradation, with t_50_ values of 15.5 and 13.65 h, respectively. **F4** at pH 1.2 also showed improved stability (t_50_ = 12.18 h), although all PEG-based formulations degraded faster at pH 1.2 than at pH 5.0. This trend is consistent with the known effect of acidic conditions accelerating hydrolytic degradation of polysaccharide-based networks [58]. Thus, acidic pH conditions debilitate GG, which is more stable at a pH range from 5.0 to 8.0. PEG_10_ formulations [**F5** (PEG_10_-AMOX_25_) and **F6** (PEG_10-_AMOX_40_)] also demonstrated moderate stability, with t_50_ values ranging from 8.56 to 9.73 h.

Comparison of porogen-containing systems showed that sucrose-IPN degraded faster than PEG-IPN, attributable to the high water solubility of sucrose (2039 g/L at 20 °C) [59], which promotes speedy water uptake, accelerating disintegration. Thus, formulations containing sucrose as porogen (p. 4, [56]) showed intermediate stability between non-porogen and PEG-based IPN. At pH 5.0, **F9** (Suc_5_-AMOX_25_) achieved a t_50_ of 12.85 h, while **F10** had t_50_ values between 4.44 and 9.37 h. At pH 1.2, sucrose-based IPN degraded more rapidly, with t_50_ values as low as 4.44 h (**F14**, Suc_10_-AMOX_40_). This suggests that while sucrose can enhance matrix stability compared to no porogen, it is less effective than PEG, particularly under acidic conditions.

Overall, the most stable formulations were those containing PEG as a porogen at pH 5.0, especially **F3** and **F4**. Sucrose-based formulations at pH 5.0 (notably **F9**) also approached the required stability for GRDDS, which demand a survival time of 12–24 h. In contrast, all formulations at pH 1.2, as well as non-porogen and most sucrose-based IPNs at pH 5.0, degraded too quickly for prolonged gastric retention. These findings highlight the critical role of both porogen type and environmental pH in designing IPN matrices for GRDDS applications, with PEG-based formulations at near-neutral pH offering the most promising degradation profiles.

It is noteworthy that the safety profile of the IPN formulation is supported by rigorous chemical methodology and thoughtful material selection. The DA reaction, with high yields and exceptional selectivity, reduces the risk of unwanted byproducts and ensures structural control in the resulting network. During the design process, monomeric and crosslinking agents were systematically evaluated against established toxicophore databases. Compounds known to introduce toxicological liabilities were excluded [60]. The principal constituents (GG, PEG, and Suc) are well-established as biocompatible and are commonly employed in pharmaceutical and biomedical settings. The solvent DMSO, utilized at carefully controlled concentrations, has a robust safety record in biomedical practice and does not pose hazards at the levels used [61]. Upon hydrogel degradation, the retro-Diels–Alder mechanism quantitatively yields the original, biocompatible monomers. This process does not produce additional functional groups or toxic byproducts. Accordingly, each phase of IPN design and synthesis, including byproduct formation, was strategically guided to preclude toxic outcomes. This ensured that the final material’s safety is firmly anchored in predictive toxicophore analysis and the known biocompatibility of all constituents.

### 2.6. Microstructure of IPN and Formulations

**Figure 5:**
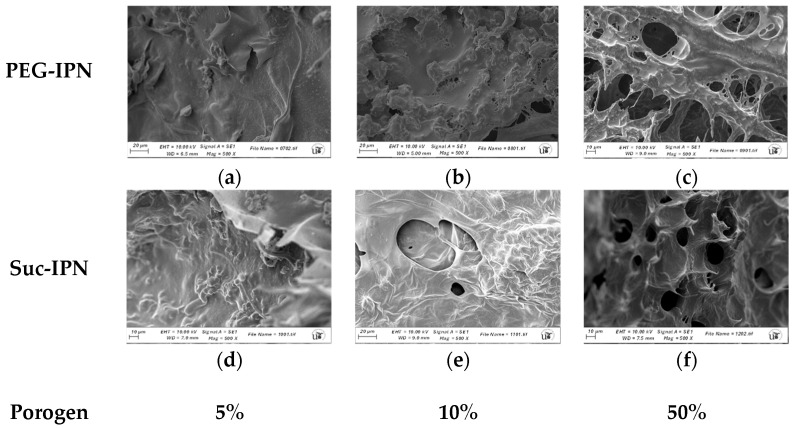
Scanning electron microscopy images (SEM, 500X magnification): (**a**) **B3.4** (GG-DA-PEG_5_); (**b**) **B5.6** (GG-DA-PEG_10_); (**c**) **B7.8** (GG-DA-PEG_50_); (**d**) **B9.10** (GG-DA-Suc_5_); (**e**) **B11.12** (GG-DA-Suc_10_); (**f**) **B13.14** (GG-DA-Suc_50_).

Morphological characterization of the systems was performed by scanning electron microscopy (SEM) to assess sample topography. The analysis confirmed that the IPN hydrogels exhibited a sponge-like microstructure, indicative of high porosity. High porosity is critical for IPN biomatrix, as it increases the exposed surface area and enhances material exchange [42], as demonstrated by AMOX release in this study. As reflected in Figure 5 and Table S1, both PEG and sucrose were effective in generating porous structures, with notable differences in pore distribution observed depending on the type and concentration of porogen. An increased concentration of pore-generating agents resulted in augmented porosity within the systems. For instance, the blank GG-DA-PEG_10_ (**B5.6**) exhibited a porous area of 6.42%, whereas the blank GG-DA-PEG_50_ (**B7.8**) demonstrated a porous area of 13.2%. Furthermore, the mean pore diameter and perimeter in the targets with higher porogen concentrations were found to be larger (ca. 1.297 μm and 4.074 μm, respectively, for **B5.6** vs. ca. 8.426 μm and 26.472 μm, respectively, for **B7.8**).

**Figure 6:**
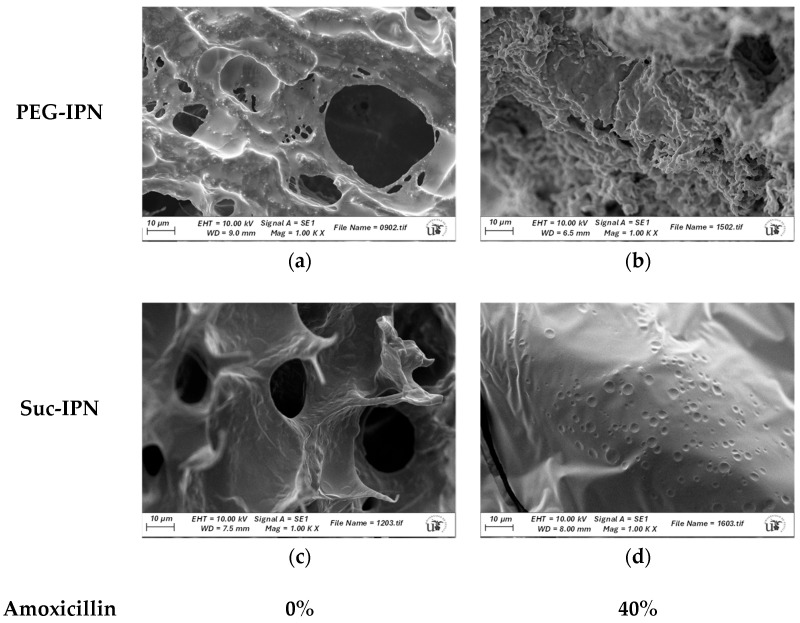
Scanning electron microscopy images (SEM, 1000× magnification): (**a**) **B7.8**: GG-DA-PEG_50_; (**b**) **F8**: GG-DA-PEG_50_-AMOX_40_; (**c**) **B13.14**: GG-DA-Suc_50_; (**d**) **F14**: GG-DA-Suc_50_-AMOX_40_.

The incorporation of AMOX into IPN systems was evaluated using SEM analysis. As can be seen in Figure 6 and Table S1, the presence of AMOX in both PEG- and sucrose-based networks resulted in a noticeable reduction in apparent porosity. For example, the mean pore perimeter in blank **B13.14** was 57.292 μm, whereas in system **F14** it was 6.260 μm. This effect may be attributed to AMOX molecules occupying pore spaces, thereby filling the voids and reducing the number of visible pores. Similar findings have been reported in the literature, where drug loading into hydrogel matrices leads to decreased porosity due to the physical occupation of pores by drug particles [51,57].

### 2.7. Kinetic Evaluation of Controlled Drug Release Using Peppas and Higuchi Models

The conducted studies focused on evaluating the release kinetics of AMOX from IPN hydrogels designed for controlled gastrointestinal delivery. Since AMOX is usually prescribed in combination with proton pump inhibitors for *H. pylori* eradication, the gastric pH increases to around 5.0, under which AMOX predominantly exists in its zwitterionic form [4]. In this context, the efficiency of the novel materials as GRDDS will be appraised at both pH 1.2 and pH 5.0, to ascertain their capacity to release AMOX in both cationic and zwitterionic forms. The main objective was to assess how key variables, such as drug loading method (pre- or post-loading), type and concentration of porogen (PEG or sucrose), and simulated medium pH (1.2 and 5.0), influence the rate and extent of AMOX release. These experiments aim to optimize sustained-release systems responsive to physiological conditions, thereby enhancing therapeutic efficacy and drug stability in the gastrointestinal tract. The release data from the formulations prepared at fixed times are recorded in the repository of the University of Seville [56].

Korsmeyer–Peppas and Higuchi mathematical models were employed to characterize the time-dependent AMOX release from IPN hydrogels in SGF (pH 1.2 and pH 5.0), providing critical insights into the underlying transport mechanisms (Tables S2–S5 from Supplementary Materials) [62]. These models enable quantitative differentiation of the release kinetics, allowing precise identification of whether drug release is governed primarily by diffusion, polymer matrix swelling, or erosion [63,64].

**Figure 7:**
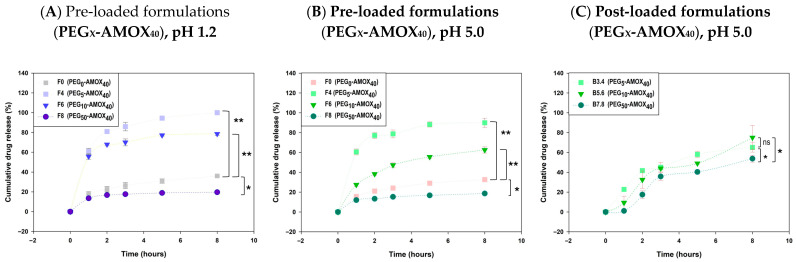
Kinetic comparison of AMOX release from IPN hydrogels: effect of PEG content, drug loading method, and pH. Each measurement was performed in triplicate (technical replicates, *n* = 3). Asterisks on the figure indicate significant differences between each IPN; *: *p* < 0.05, **: *p* < 0.01, and ns: not significant.

Pre-loaded systems demonstrated a stronger alignment with kinetic models than post-loaded systems, as evidenced by consistently higher R^2^ values. For example, AMOX release from pre-loaded PEG-based formulations (**F3–F8**) exhibited superior release control compared with post-loaded analogues (Tables S2–S5), supporting the reliability of the one-pot strategy that integrates matrix formation with drug loading. The Korsmeyer–Peppas model provided the optimal fit, with R^2^ values generally above 0.90 at pH 1.2 and for most formulations at pH 5.0, with the exception of sucrose-rich systems with high drug content (**F12–F14**). Diffusion exponents from this model were typically less than 0.45 in pre-loaded systems, confirming a diffusion-controlled release mechanism. These findings underscore the efficacy of the Korsmeyer–Peppas model in describing drug release from AMOX pre-loaded IPN hydrogels [63]. The cumulative AMOX release after 8 h in PEG-containing pre-loaded formulations ranged from 18.64% (**F8**, PEG_50_-AMOX_40_, pH 5.0) to 100% (**F4**, PEG_5_-AMOX_40_, pH 1.2), demonstrating the strong influence of PEG content and initial drug loading on release behavior (pp. 5 and 7 [56]). Formulations with higher drug loading (40%, **F4**, **F6**, **F8**) exhibited the most pronounced differences in release kinetics (Figure 7A,B). Increasing the PEG concentration from 5% to 50% resulted in a progressive reduction in release rate. At a pH of 1.2, complete release was achieved in **F4** (PEG_5-_AMOX_40_) within 8 h, compared with 78.59% and 19.63% for **F6** (PEG_10_-AMOX_40_) and **F8** (PEG_50_-AMOX_40_), respectively. A similar trend was observed at pH 5.0, with 8 h release values of 89.99% (**F4**), 62.54% (**F6**), and 18.64% (**F8**).

The higher release observed in low PEG-content formulations (**F4** and **F6**, 5–10% PEG) compared to PEG-free controls can be attributed to the formation of interconnected porous network that promote rapid matrix hydration and facilitate AMOX diffusion. Such structures also explain the pronounced burst release observed in these systems (Figure 7A,B). Conversely, formulations with high PEG content (e.g., **F8**, 50%) exhibited markedly reduced release, even lower than PEG-free systems. At these concentrations, PEG chains form extensive entanglements with GG and the crosslinked DA polymer, producing dense microstructured networks stabilized by hydrogen bonding and ion–dipole interactions. This process reinforces the IPN structure (see Rheological Analysis, Section 2.2). These interactions restrict water influx and drug mobility, leading to compact matrices that strongly retain AMOX. This results in suppressed burst release and significantly limited long-term diffusion pathways, consistent with previous works [65].

In post-loaded AMOX formulations, the PEG-based systems showed a gradual release of AMOX over time; however, their release profiles exhibited suboptimal fit to release models, as indicated by lower R^2^ values and greater variability. The only post-loaded PEG formulation that adequately fit a kinetic model (Higuchi) was the counterpart of pre-loaded **F5** (PEG_10_–AMOX_25_) at both pH values (Tables S2 and S3). When AMOX was loaded at 40%, all formulations displayed convergent kinetics at pH 5.0 (Figure 7C), suggesting that the porogenic effect of PEG is diminished during post-loading due to incomplete AMOX penetration and drug saturation at the bead surface, which accelerates release. In high-PEG systems (50%), a delayed onset was observed, likely due to restricted fluid ingress, followed by faster release after two hours consistent with bead swelling and matrix relaxation.

**Figure 8:**
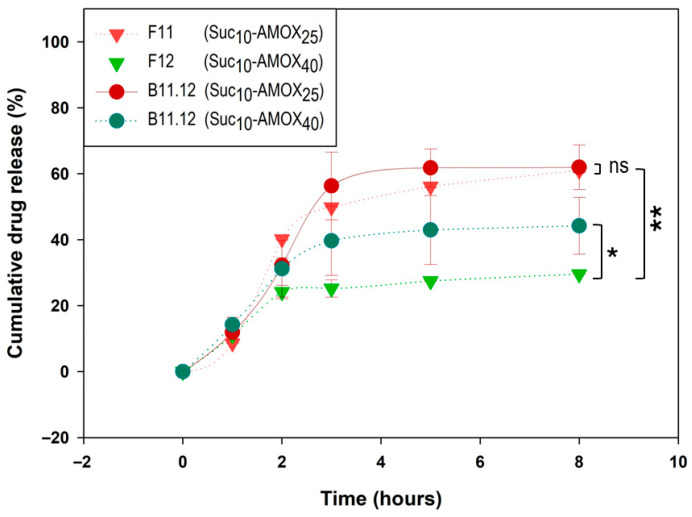
Comparative kinetic profiles of AMOX release in SFG (pH 5.0) from IPN hydrogels with 10% sucrose porogen: influence of pre- and post-loading and initial AMOX content (25% vs. 40%). The measurement was performed in triplicate (technical replicates, *n* = 3). Asterisks on the figure indicate significant differences between each IPN; *: *p* < 0.05, **: *p* < 0.01, and ns: not significant.

For post-loaded sucrose-based IPNs, Suc_10_ formulations provided the most gradual AMOX release, particularly at high drug loading. The Suc_10_-AMOX_40_ post-loaded system (**B11.12**, pH 5.0) showed a delayed onset followed by gradual release, reaching 44.22% at 8 h (Figure 8). In comparison, the corresponding pre-loaded formulation (**F12**, Suc_10_-AMOX_40_, pH 5.0) released 29.58% at 8 h, indicating slower release in pre-loaded systems.

Although formulations with low sucrose content (Suc_5_) exhibited pronounced burst release—for example, **F9** (Suc_5_-AMOX_25_, pH 5.0) released 45.97% at 1 h (p. 6, [56]), and post-loaded counterpart **B9.10-AMOX_25_** (pH 5.0) released 59.43% at 1 h (p. 8, [56])—they were also the sucrose-based systems that best fitted the Korsmeyer–Peppas model (Tables S3 and S5).

Conversely, high sucrose content (Suc_50_) in pre-loaded systems (**F13**, **F14**) did not further reduce release as seen with PEG; instead, Suc_50_ profiles overlapped with Suc_5_, likely due to bead destabilization and rapid matrix disintegration.

At high AMOX loading (40%), pre-loaded Sucrose IPN (**F10**, **F12**, **F14**) exhibited similar and low release at pH 5.0 (**F10**: 21.88%, **F12**: 29.58%, **F14**: 23.26% at 8 h), indicating that porogen content had a less pronounced effect than in PEG systems. For lower AMOX loading (25%), the effect of sucrose concentration was minimal, with release profiles converging for Suc_10_ and Suc_50_.

In summary, pre-loaded PEG IPNs enable better control of AMOX release, with higher PEG concentrations effectively minimizing burst release and slowing overall release rates. In contrast, post-loaded PEG IPNs display greater variability and faster release, particularly at pH 5.0, likely due to limited drug penetration and surface saturation. Sucrose-based IPNs show distinct behavior: post-loaded Suc_10_ systems provide moderate, sustained release, while Suc_5_ and Suc_50_ formulations are susceptible to burst release and bead instability. At high AMOX loading, sucrose concentration has less impact on release kinetics than PEG, especially at the higher pH tested.

Overall, as shown in Tables S6–S13, the AMOX concentration released from prepared systems consistently exceeds the MIC_50_ (0.023 mg/mL) and frequently exceeds the MIC_90_ (0.125 mg/mL) in almost all the time slots [7]. This ensures the desired antibiotic effect against *H. pylori* infections from the first hour of administration, even at the lowest release rate.

The results of this study demonstrate the significance of uniform encapsulation and controlled porogen content in minimizing burst release and achieving sustained, predictable profiles [66].

## 3. Conclusions

This work reports, for the first time, the one-step synthesis of guar gum-based interpenetrating polymer network (IPN) hydrogels, in which the Diels–Alder crosslinking process and amoxicillin (AMOX) loading occur simultaneously under mild conditions that do not affect the chemical integrity of the antibiotic. The process involves 21 formulations that were systematically varied, including 14 that were loaded with either 25% or 40% AMOX (*w*/*w*, relative to the polymer mass). It employs biocompatible porogens (PEG or sucrose at 5%, 10%, or 50% *w*/*w*) that are incorporated directly during IPN formation. Unlike the controls lacking Polymer 2, all hydrogels maintained homogeneous, stable gel-like matrices, accentuating the necessity of both crosslinking and strategic porogen inclusion for mechanical and functional stability.

All AMOX pre-loaded IPN systems (**F1–F14**) and unloaded reference matrices (**B1.2**-**B13.14**) exhibited suitable rheological properties, including gel-like behavior, mechanical strength, and stability. For example, 10% PEG increases the swelling index (SI) to 2002%, quadrupling that of the base GG-DA IPN (SI = 497%), while supporting the greatest mechanical robustness. Storage modulus ($G^{\prime }$) reaches 10,054 Pa, and complex viscosity is up to 1302.3 Pa·s at 50% PEG and 40% AMOX loading (**F8**). In contrast, 50% sucrose drives the SI to 1895% and the critical strain (${\dot{\gamma }}_{c}$) above 20%, generating highly ductile, readily swellable matrices.

We systematically evaluated the drug delivery performance of the synthesized IPN hydrogels, focusing on the influence of porogen type, loading strategy, and AMOX concentration. All AMOX-loaded networks exhibited robust encapsulation and the presence and concentration of porogens were critical for optimizing release kinetics. PEG-based IPNs exhibited prolonged and controlled AMOX delivery, while maintaining matrix integrity and buoyancy for over 24 h. In contrast, sucrose-based IPNs showed accelerated drug release and matrix disintegration, especially at high concentrations or the most acidic pH. The most effective systems, characterized by swelling properties (swelling index > 500%), sustained buoyancy (>12 h), high relative stability (t_50_ > 8.5 h), and controlled drug release that fits predominantly the Korsmeyer–Peppas model (or, alternatively, the Higuchi model with R^2^ > 0.90) at both pH 1.2 and 5.0, were as follows: (a) pre-loaded one-pot systems: **F3** and **F4** (PEG_5_–AMOX_25/40_), **F5** and **F6** (PEG_10_-AMOX_25/40_), and **F10** (Suc_5-_AMOX_40_); and (b) post-loaded systems equivalent to **F5** (PEG_10_–AMOX_25_), **F9**, and **F10** (Suc_5_–AMOX_25/40_).

For gastroretentive drug delivery applications, pre-loaded IPNs are preferred over post-loaded ones, and PEG is the recommended porogen over sucrose. Optimal porogen concentrations are 5–10% for PEG and 5% for sucrose. The release rate can be adjusted between 25% and 40% to ensure precise control over the drug loading. In all cases, the formulations provided drug concentrations that exceeded the MIC for *H. pylori* throughout the release period, assuming gastric replenishment of 1 L per hour.

## 4. Materials and Methods

### 4.1. Materials

The chemicals utilized in this work were purchased from Sigma-Aldrich and used as received: gum guar (GG), methoxypolyethylene glycol 350 (PEG), amoxicillin (AMOX) and sucrose (Suc).

### 4.2. IPN Preparation

Twenty-one IPNs were synthesized (Table 6, Table 7 and Table 8), comprising seven blanks (**Bn.m**) and fourteen AMOX-loaded formulations (**F1**–**F14**). All hydrogels were prepared by polymerizing intertwined Polymer 2 (DA network, targeting 10 mol% of crosslinking) within a colloidal solution of GG (Polymer 1), with each polymer present at a concentration of 4% *w*/*v*. To enhance porosity, PEG (Table 7) or sucrose (Table 8) were employed as a porogen. This experimental design enabled systematic evaluation of the influence of porogens on IPN structure and function. AMOX was incorporated into selected formulations mass (**F1**–**F14**, Table 6, Table 7 and Table 8) at 25% or 40% *w*/*w* relative to total polymer mass. The addition of AMOX was performed during the formation of the IPNs. Blanks are listed first on each table and designated as **Bn.m,** where ‘*n*’ and ‘*m*’ correspond to the associated AMOX-loaded formulations. For example, **B1.2** (Table 6) serves as the blank for formulations **F1** and **F2**, which differ only in AMOX content (25% and 40%, respectively).

For IPN preparation, monomers and crosslinker were dissolved in the minimal amount of DMSO and added to the colloidal solution of GG to achieve a theoretical crosslinking density of 10%. Depending on the blank or formulation, a porogen (PEG or sucrose) was incorporated at concentrations of 5, 10, or 50% *w*/*w*. For AMOX-loaded systems, AMOX was dissolved in DMSO (concentration: 0.5 g/mL) and introduced during the one-pot synthesis to obtain hydrogels containing 25% or 40% *w*/*w* AMOX. For illustrative purposes, the preparation of the IPN GG-DA-AMOX_25_ (**F1**) is described next: GG (200 mg) was dissolved in 4.0 mL of distilled water to obtain a homogeneous colloidal dispersion. Additionally, the monomers and crosslinker were dissolved in DMSO at the following concentrations: Di-Fur (89.8 mg in 160 μL DMSO, 561.3 mg/mL^−1^), Tri-Fur (11.2 mg in 60 μL DMSO, 186.7 mg/mL^−1^), and Di-Mal (99.8 mg in 226 μL DMSO, 441.6 mg/mL^−1^). Amoxicillin (AMOX, 133.3 mg) was dissolved in 0.267 mL of DMSO (500 mg/mL). The final volume was adjusted to 5.00 mL by adding sufficient distilled water, ensuring that both the DA polymeric network and the GG concentrations of 4.00% *w*/*v* in the reaction medium were met. The mixture was stirred in the absence of light for 48 h and then stored in the dark to prevent AMOX degradation due to light exposure [67].

The IPN nomenclature (**GG-DA-Por_x_-AMOX_y_**) is defined as follows: “**GG**” indicates the inclusion of guar gum in the polymer matrix; “**DA**” denotes Polymer 2, a Diels–Alder crosslinked network. “**Por_x_**” specifies the presence and type of porogen—either “**PEG_x_**” for PEG-350 or “**Suc_x_**” for sucrose—where “x” represents the porogen concentration (5%, 10%, or 50% *w*/*w*). “**AMOX_y_**” indicates the AMOX content (25% or 40% *w*/*w*); this suffix is omitted in the blanks. The corresponding systems are detailed in Table 6, Table 7 and Table 8.

The general preparation procedure is exemplified by **F4** (**GG-DA-PEG_5_-AMOX_40_**). In a vial, a colloidal solution of GG was prepared by dissolving 200 mg of GG in 3.5 mL of H_2_O. Monomers and crosslinker, pre-dissolved in DMSO, were sequentially added as follows: Di-Fur [45] (90 mg in 150 μL DMSO), Tri-Fur (11 mg in 60 μL DMSO), and Di-Mal [46] (100 mg in 226 μL DMSO). PEG-350 (21 mg) and AMOX (266.6 mg, dissolved in DMSO at 0.5 g/mL) were then incorporated, and the total volume was adjusted to 5 mL with water. The mixture was manually stirred for 5 min to ensure homogeneity, followed by 48 h of mixing on a roller at room temperature. This protocol was applied to all IPN formulations, with specific component variations detailed in Table 6, Table 7 and Table 8. For sucrose-based systems, sucrose was pre-dissolved in water at 0.5 g/mL.

After 48 h, the rheological properties of the IPNs were measured. The samples were subsequently lyophilized for further characterization, including floatability, degradation, swelling, and controlled drug release studies.

### 4.3. General Methods

Experiments of rheology and scanning electron microscopy (SEM) were conducted at the CITIUS facilities of the Universidad de Sevilla.

The rheological measurements of the hydrogels were performed in a Discovery HR-3 (TA Instruments, New Castle, DE, USA) rheometer equipped with a Peltier temperature controller at 25 °C using a plate–plate geometry (diameter: 40 mm). The linear viscoelasticity range was performed at a frequency of 2π rad∙s through strain amplitude sweeps. At least two replicates were performed on fresh samples. Lyophilization of the IPN was also carried out using CITIUS facilities, to preserve its porous structure and avoid its deformation or degradation that could occur during a natural drying process, ensuring the stability of the samples. Images were recorded specifically at the Microscopy Laboratories by means of a field emission scanning electron microscope, Zeiss EVO (Carl Zeis, Oberkochen, Germany), at an accelerating voltage of 10 kV using secondary electrons.

Absorbance measures required for AMOX calibration curve and release studies were conducted using a UV-1280 Multipurpose UV–Visible Spectrophotometer (Shimadzu Corporation, Kyoto, Japan).

Data analysis was performed using Microsoft Office Excel 365. Unless stated otherwise, all experiments were conducted in triplicate, and the results are expressed as the mean ± standard deviation (SD). Statistical significance was assessed using *t*-Test calculator (https://www.graphpad.com/quickcalcs/ttest1/ accessed on 2 June 2025), considering *p* < 0.05 as the threshold for significance.

### 4.4. Characterization of Guar-Gum-Based IPN

A. Rheological properties

The rheological properties of the hydrogels were assessed using both strain and frequency sweep tests at 25 °C. Initially, strain sweep measurements (0.02–100% strain at a fixed angular frequency of 2π rad·s) were performed to determine the linear viscoelastic range and the critical strain (${\dot{\gamma }}_{c}$) of each sample. Subsequently, frequency sweep tests (1 × 10^−1^ to 7 × 10^2^ rad/s) were conducted at a constant strain within the linear viscoelastic range to evaluate the stability of the samples over a range of frequencies. These analyses yielded key rheological parameters, including the storage modulus ($G^{\prime }$), loss modulus ($G^{\prime \prime }$), their ratio (tan (δ) = G″/G′), the complex viscosity (η*) and the *plateau* modulus ($G_{N}^{0}$).

B. Swelling properties

The swelling index (SI) was determined for the samples **B1.2**, **B3.4**, **B5.6**, **B7.8**, **B9.10**, **B11.12**, and **B13.14**. To investigate swelling kinetics, two 20 mg samples of each IPN were weighed and placed in a 5 mL Eppendorf tubes at room temperature. Distilled water was added incrementally until visual saturation was achieved. The swollen IPNs were then reweighed, and the final mass ($W_{2}$) was compared to the initial mass ($W_{1}$), to calculate the *SI* according to Equation (1).

(1) $$SI=\frac{{(W}_{2}-W_{1})}{W_{1}}\times 100$$

This index represents the number of times the hydrogel can absorb its own dry weight in water, expressed as a percentage. It quantitatively reflects the hydrogel’s water absorption capacity.

C. Microstructural analysis of the systems (SEM)

The topography morphology of the IPN was studied by scanning electron microscopy (SEM) on the following lyophilized samples: **B3.4**, **B5.6**, **B7.8**, **F3**, **F8**, **B9.10**, **B11.12**, **B13.14**, **F9**, **F14**. The porosity of the systems was evaluated from the resulting SEM images, with the use of a digital processing free software: FIJI Image-J (Image-J 1.54p, National Institutes of Health, Bethesda, MD, USA) [68]. The SEM images were then subjected to image thresholding using Otsu’s method, a widely recognized technique for optimal image segmentation [69]. This approach facilitated the objective differentiation between the porous regions and the hydrogel matrix, thereby enabling a more precise characterization of the material’s microstructure.

D. Degradation kinetics and floatability of AMOX-loaded IPNs

For each formulation, the time (in hours) to reach 50% degradation of formulation mass was determined using the experimental data obtained from the degradation assays performed at both pH 1.2 and pH 5.0. In these assays, the percentage of remaining IPN mass was measured at predetermined time intervals under simulated physiological conditions (37 °C, 150 rpm). The resulting mass loss data (p. 4, [56]) were plotted as a function of time for each formulation and pH condition.

To accurately describe the degradation kinetics, the experimental data were fitted to logarithmic trend curves. All formulations exhibited a logarithmic degradation profile, with coefficients of determination (R^2^) exceeding 0.95, indicating an excellent fit to the model. The time to 50% degradation of formulation mass was then calculated from these fitted curves as the time point at which 50% of the initial mass remained.

All degradation experiments were performed in triplicate to ensure reproducibility and reliability of the results. The values reported in the table represent the mean values obtained from these independent experiments.

Floatability properties were assessed during release assays. Both the floating lag time (FLT) and total floating time (FT) were determined by empirical visual observation.

### 4.5. Controlled Drug Release Studies

A. AMOX Loading and Release Assays

AMOX loading into the IPN was performed using two distinct methods. In the first approach, a one-pot procedure, the IPN was formed in situ while the drug was added to the medium during network formation (**F1–F14**). In the second method, AMOX was dissolved in DMSO (0.5 mg/mL) and subsequently loaded onto a pre-formed IPN (in all Blanks). These two loading strategies were designed to investigate the influence of the loading process on drug release behavior.

In these experiments, the controlled drug release capability of the three-dimensional IPN was assessed using freeze-dried AMOX-loaded IPN samples at pH 1.2 (SGF-2) and pH 5.0 (SGF-5). For each formulation, three lyophilized beads (50 mg each) were placed in a beaker containing 150 mL of the corresponding fresh medium and incubated at 37 °C with continuous stirring at 100 rpm. At predeterminate time intervals (1 h, 2 h, 3 h, 5 h, 8 h and 24 h), 3 mL aliquots of the incubation medium were withdrawn, and their absorbance was measured using a UV spectrophotometer at the maximum absorbance wavelength of AMOX (229 nm). The cumulative release of AMOX was quantified using a calibration curve generated from standard solutions in the concentration range of 3.5 × 10^−1^ to 5 × 10^−3^ mg/mL. Equation (2) relates absorbance (*y*) to AMOX concentration (*x*, mmol·mL^−1^), with the linear correlation coefficient (*R*^2^) indicating the quality of fit. Samples were diluted as necessary to ensure absorbance readings remained within the calibration range.

(2) $$y=9084.2\;x+0.0072\;\left(R^{2}=0.9994\right)$$

In order to assess the calculation of the AMOX concentration that could be achieved in the stomach at each interval and to be able to compare it with the MIC, a maximum stomach volume of 1.7 L was considered [70]. The AMOX concentration for each interval was determined using Equation (3) and is displayed in Tables S6–S13.

(3) $$[AMOX]=\frac{W_{AMOX}}{1.7}$$

where $W_{AMOX}$ denotes the quantity of AMOX (mg) released in each time slot. These values were calculated based on the cumulative release of AMOX, considering that samples loaded with 25% *w*/*w* AMOX contain a maximum of 33.3 mg of drug and those loaded with 40% *w*/*w* contain an initial total of 66.6 mg of AMOX.

B. Kinetics modeling of drug release

The release profiles AMOX from the prepared formulations were analyzed using Korsmeyer–Peppas and Higuchi mathematical models. Model fitting to the experimental data was performed using OriginPro 9.0 software (OriginLab Corporation, Northampton, MA, USA), with the goodness of fit evaluated by the coefficient of determination (R^2^).

The data were evaluated according to the following equations:

(4) Higuchimodel    Qt = kH·t12

where $Q_{t}$ is the amount of drug released at time *t*; $k_{H}$ is the release rate constant for the Higuchi model.

(5) KorsmeyerPeppasmodel: MtM∞=kKP·tn

where MtM∞ is the fraction of drug released at time *t*; $k_{KP}$ is the rate constant; n is the release exponent, indicative of the mechanism of drug release. The first 60% of drug release data was fitted in Higuchi and Korsmeyer–Peppas model.

## Figures and Tables

**Table 1:** Rheological properties of simple guar gum colloidal dispersions.

KEY COMPOSITION	${\dot{\gamma }}_{c}\;(\%)$	$G_{1}^{\prime }\;(Pa)$	$G_{1}^{\prime \prime }\;(Pa)$	*tan* (δ)	$\eta_{1}^{*}\;(Pa\cdot s)$	$G_{N}^{0}\;\left(Pa\right)$
**GG**	0.51	489	101	0.21	79.3	888
**GG-PEG_5_**	8.08	929	321	0.35	124.0	1346
**GG-PEG_10_**	5.10	874	309	0.35	116.9	1277
**GG-PEG_50_**	2.07	1545	355	0.23	200.5	1990
**GG-Suc_5_**	8.11	1124	374	0.33	149.4	1605
**GG-Suc_10_**	8.11	1089	360	0.33	144.7	1552
**GG-Suc_50_**	12.91	1002	362	0.36	134.3	1476

${\dot{\gamma }}_{c}$: critical strain; $G_{1}^{\prime }$: elastic modulus at 2π rad∙s; $G_{1}^{\prime \prime }$: viscous modulus at 2π rad∙s; **tan** (**δ**): loss tangent; $\eta_{1}^{*}$: complex viscosity at 2π rad∙s; $G_{N}^{0}$: plateau modulus; **GG**: guar gum; **PEG**: porogen polyethylene glycol-350; **Suc**: porogen sucrose.

**Table 2:** Rheological properties of non-drug-loaded IPN hydrogels.

NAME	KEY COMPOSITION	${\dot{\gamma }}_{c}\;(\%)$	$G_{1}^{\prime }\;(Pa)$	$G_{1}^{\prime \prime }\;(Pa)$	*tan* (δ)	$\eta_{1}^{*}\;(Pa\cdot s)$	$G_{N}^{0}\;\left(Pa\right)$
**B1.2**	**GG-DA**	1.01	1227	476	0.39	208.9	2117
**B3.4**	**GG-DA-PEG_5_**	5.11	1214	441	0.37	163.0	1811
**B5.6**	**GG-DA-PEG_10_**	5.10	1026	384	0.37	138.2	1544
**B7.8**	**GG-DA-PEG_50_**	5.13	959	319	0.35	123.4	1351
**B9.10**	**GG-DA-Suc_5_**	8.13	925	360	0.38	129.2	1440
**B11.12**	**GG-DA-Suc_10_**	12.93	982	371	0.38	132.4	1486
**B13.14**	**GG-DA-Suc_50_**	20.60	994	360	0.36	133.3	1474

${\dot{\gamma }}_{c}$: critical strain; $G_{1}^{\prime }$: elastic modulus at 2π rad∙s; $G_{1}^{\prime \prime }$: viscous modulus at 2π rad∙s; **tan** (**δ**): loss tangent; $\eta_{1}^{*}$: complex viscosity at 2π rad∙s; $G_{N}^{0}$: plateau modulus; **GG**: guar gum; **PEG**: porogen polyethylene glycol-350; **Suc**: porogen sucrose.

**Table 3:** Rheological properties of AMOX-loaded hydrogels.

NAME	KEY COMPOSITION	${\dot{\gamma }}_{c}\;(\%)$	$G_{1}^{\prime }\;(Pa)$	$G_{1}^{\prime \prime }\;(Pa)$	*tan* (δ)	$\eta_{1}^{*}\;(Pa\cdot s)$	$G_{N}^{0}\;\left(Pa\right)$
**F1**	**GG-DA-AMOX_25_**	1.00	887	201	0.23	120.8	1085
**F2**	**GG-DA-AMOX_40_**	5.09	1554	376	0.24	253.8	2751
**F3**	**GG-DA-PEG_5_-AMOX_25_**	3.23	2252	718	0.32	298.1	2976
**F4**	**GG-DA-PEG_5_-AMOX_40_**	0.52	4657	1399	0.30	613.3	6112
**F5**	**GG-DA-PEG_10_-AMOX_25_**	8.28	1357	458	0.34	180.6	1947
**F6**	**GG-DA-PEG_10_-AMOX_40_**	0.33	4259	1269	0.30	560.5	5587
**F7**	**GG-DA-PEG_50_-AMOX_25_**	3.26	1441	407	0.28	188.8	1970
**F8**	**GG-DA-PEG_50_-AMOX_40_**	0.51	10,054	2351	0.23	1302.3	12,702
**F9**	**GG-DA-Suc_5_-AMOX_25_**	12.87	890	354	0.40	120.8	1368
**F10**	**GG-DA-Suc_5_-AMOX_40_**	8.13	834	274	0.33	110.8	1198
**F11**	**GG-DA-Suc_10_-AMOX_25_**	8.07	1079	361	0.33	143.5	1548
**F12**	**GG-DA-Suc_10_-AMOX_40_**	20.95	897	289	0.32	118.9	1276
**F13**	**GG-DA-Suc_50_-AMOX_25_**	8.09	1070	357	0.33	142.2	1535
**F14**	**GG-DA-Suc_50_-AMOX_40_**	21.12	1043	312	0.30	137.3	1442

${\dot{\gamma }}_{c}$: critical strain; $G_{1}^{\prime }$: elastic modulus at 2π rad∙s; $G_{1}^{\prime \prime }$: viscous modulus at 2π rad∙s; **tan** (**δ**): loss tangent; $\eta_{1}^{*}$: complex viscosity at 2π rad∙s; $G_{N}^{0}$: plateau modulus; **AMOX**: amoxicillin; **GG**: guar gum; **PEG**: porogen polyethylene glycol-350; **Suc**: porogen sucrose.

**Table 4:** Comparative analysis of rheological parameters for IPN hydrogels with different porogen types and AMOX content regarding GG colloidal dispersions or hydrogels.

System Type	Porogen	AMOX	${\dot{\gamma }}_{c}$	$G_{1}^{\prime }$	$G_{N}^{0}$	tan (δ)	$\eta_{1}^{*}$	Key Trend
GG only	None	0	0.51%	489 Pa	888 Pa	0.21	79.3 Pa·s	Weak, soft gels
GG + PEG	PEG	0	**↑**	**↑**	**↑**	**~**	**↑**	Stiffer, more elastic with ↑ PEG
			(up to 2.44%)	(up to 1545 Pa)	(up to 1990 Pa)		(up to 186 Pa·s)	
GG + Suc	Sucrose	0	**↑↑**	**↑**	**↑**	**~**	**↑**	More flexible, less stiff than PEG
			(up to 12.91%)	(up to 1123 Pa)	(up to 1667 Pa)		(up to 164 Pa·s)	
IPN Blank	None	0	1.01%	1227 Pa	2117 Pa	0.39	209 Pa·s	Crosslinked, improved strength
IPN + PEG	PEG	0	**↑**	**↓**	**↓**	**↓**	**↓**	Softer, more flexible; highest stiffness at high PEG
			(to ~5.1%)	(to 959 Pa)	(to 1351 Pa)	(to 0.35)	(to 123 Pa·s)	
IPN + Suc	Sucrose	0	**↑↑**	**~**	**~**	**~**	**~**	Highest flexibility at high sucrose
			(to 20.60%)	(925–994 Pa)	(1440–1474 Pa)	(0.36–0.38)	(129–133 Pa·s)	
IPN + PEG + AMOX	PEG	**√**	**↑**	**↑↑↑**	**↑↑↑**	**↓**	**↑↑**	Synergistic reinforcement at high AMOX and PEG
			(to 5.09%)	(up to 10,054 Pa)	(up to 12,702 Pa)	(0.23–0.34)	(up to 1302 Pa·s)	
IPN + Suc + AMOX	Sucrose	**√**	**↑↑**	**~**	**~**	**↑**	**~**	Flexibility increases with AMOX, less stiff
			(to 21.12%)	(up to 1043 Pa)	(up to 1489 Pa)	(up to 0.40)	(up to 137 Pa·s)	

↑: Increase compared to previous system; ↑↑: strong increase; ↑↑↑: very strong increase; ↓: decrease; ~: no significant change. ${\dot{\gamma }}_{c}$: critical strain; $G_{1}^{\prime }$: elastic modulus at 2π rad∙s; $G_{1}^{\prime \prime }$: viscous modulus at 2π rad∙s; tan (δ): loss tangent; $\eta_{1}^{*}$: complex viscosity at 2π rad∙s; $G_{N}^{0}$: plateau modulus; **AMOX**: amoxicillin; **GG**: guar gum; **PEG**: porogen polyethylene glycol-350; **Suc**: porogen sucrose.

**Table 5:** Swelling study for non-loaded IPN. Swelling index calculated from Equation (1).

NAME	KEY COMPOSITION	SI (%)	AVERAGE SI (%)	ERROR (±)	ERROR (%)
**B1.2_a**	**GG-DA**	517.9	497	21	4.1
**B1.2_b**		476.9			
**B3.4_a**	**GG-DA-PEG_5_**	1592	1585	8	0.5
**B3.4_b**		1577			
**B5.6_a**	**GG-DA-PEG_10_**	2148	2002	147	7.3
**B5.6_b**		1855			
**B7.8_a**	**GG-DA-PEG_50_**	1492	1412	80	5.7
**B7.8_b**		1332			
**B9.10_a**	**GG-DA-Suc_5_**	1738	1678	61	3.6
**B9.10_b**		1617			
**B11.12_a**	**GG-DA-Suc_10_**	2035	2058	23	1.1
**B11.12_b**		2080			
**B13.14_a**	**GG-DA-Suc_50_**	1960	1895	66	3.5
**B13.14_b**		1829			

**SI**: swelling index; **GG**: guar gum; **PEG**: porogen polyethylene glycol-350; **Suc**: porogen sucrose.

**Table 6:** Composition and identifiers of IPNs produced without porogens.

NAME	KEY COMPOSITION	IPN					AMOX	
		Polymer 1	Polymer 2					
		GG	Di-Fur	Di-Mal	Tri-Fur ^(a)^		mg	% (*w*/*w*)
		Mg	mg	mg	Mg	mol (%)		
**B1.2**	**GG-DA**	200	89.8	99.8	11.2	10	------	------
**F1**	**GG-DA-AMOX_25_**	200	89.8	99.8	11.2	10	133.3	25
**F2**	**GG-DA-AMOX_40_**	200	89.8	99.8	11.2	10	266.6	40

^(a)^ Sufficient quantity to react with 10% of the maleimide groups from Di-Mal. Experimental conditions for IPN formation: Polymer concentration 4% (*w*/*w*); Solvent: H_2_O and DMSO; crosslinker: Tri-Fur. **AMOX**: amoxicillin; **Di-Mal** (monomer): 1,8-dimaleimide-3,6-dioxaoctane; **GG**: guar gum; **IPN**: interpenetrating polymer network; **Di-Fur** (monomer): *N*,*N*’-difurfuryl-L-tartaramide; **Tri-Fur** (crosslinker): 2,3,5-tri-*O*-{[(furan-2-ylmethyl)carbamoyl]}-D-ribono-1,4-lactone.

**Table 7:** Composition and identifiers of IPNs produced with PEG-350 as porogen.

NAME	KEY COMPOSITION	IPN					AMOX		PEG
		Polymer 1	Polymer 2						
		GG	Di-Fur	Di-Mal	Tri-Fur ^(a)^		mg	% (*w*/*w*)	% (*w*/*w*)
		mg	mg	mg	mg	mol (%)			
**B3.4**	**GG-DA-PEG_5_**	300	134.7	149.7	16.8	10	------	------	5
**B5.6**	**GG-DA-PEG_10_**	300	134.7	149.7	16.8	10	------	------	10
**B7.8**	**GG-DA-PEG_50_**	300	134.7	149.7	16.8	10	------	------	50
**F3**	**GG-DA-PEG_5_-AMOX_25_**	200	89.8	99.8	11.2	10	133.3	25	5
**F4**	**GG-DA-PEG_5_-AMOX_40_**	200	89.8	99.8	11.2	10	266.6	40	5
**F5**	**GG-DA-PEG_10_-AMOX_25_**	200	89.8	99.8	11.2	10	133.3	25	10
**F6**	**GG-DA-PEG_10_-AMOX_40_**	200	89.8	99.8	11.2	10	266.6	40	10
**F7**	**GG-DA-PEG_50_-AMOX_25_**	200	89.8	99.8	11.2	10	133.3	25	50
**F8**	**GG-DA-PEG_50_-AMOX_40_**	200	89.8	99.8	11.2	10	266.6	40	50

^(a)^ Sufficient quantity to provide furfuryl rings capable of reacting with 10% of the maleimide groups present in Di-Mal. Experimental conditions for IPN formation: Polymer concentration 4% (*w*/*w*); Solvent: H_2_O and DMSO; crosslinker: Tri-Fur. AMOX added dissolved in DMSO (AMOX conc.: 0.5 g/mL). **AMOX**: amoxicillin; **Di-Mal** (monomer): 1,8-dimaleimide-3,6-dioxaoctane; **GG**: guar gum; **IPN**: interpenetrating polymer network; **PEG**: porogen polyethylene glycol-350; **Di-Fur** (monomer): *N*,*N*’-difurfuryl-L-tartaramide; **Tri-Fur** (crosslinker): 2,3,5-tri-*O*-{[(furan-2-ylmethyl)carbamoyl]}-D-ribono-1,4-lactone.

**Table 8:** Composition and identifiers of IPNs produced with sucrose as porogen.

NAME	KEY COMPOSITION	IPN					AMOX		Sucrose
		Polymer 1	Polymer 2						
		GG	Di-Fur	Di-Mal	Tri-Fur ^(a)^		mg	% (*w*/*w*)	% (*w*/*w*)
		mg	mg	mg	mg	mol (%)			
**B9.10**	**GG-DA-Suc_5_**	300	134.7	149.7	16.8	10	------	------	5
**B11.12**	**GG-DA-Suc_10_**	300	134.7	149.7	16.8	10	------	------	10
**B13.14**	**GG-DA-Suc_50_**	300	134.7	149.7	16.8	10	------	------	50
**F9**	**GG-DA-Suc_5_-AMOX_25_**	200	89.8	99.8	11.2	10	133.3	25	5
**F10**	**GG-DA-Suc_5_-AMOX_40_**	200	89.8	99.8	11.2	10	266.6	40	5
**F11**	**GG-DA-Suc_10_-AMOX_25_**	200	89.8	99.8	11.2	10	133.3	25	10
**F12**	**GG-DA-Suc_10_-AMOX_40_**	200	89.8	99.8	11.2	10	266.6	40	10
**F13**	**GG-DA-Suc_50_-AMOX_25_**	200	89.8	99.8	11.2	10	133.3	25	50
**F14**	**GG-DA-Suc_50_-AMOX_40_**	200	89.8	99.8	11.2	10	266.6	40	50

^(a)^ Sufficient quantity to provide furfuryl rings capable of reacting with 10% of the maleimide groups present in Di-Mal. Experimental conditions for IPN formation: Polymer concentration 4% (*w*/*w*); Solvent: H_2_O and DMSO; crosslinker: Tri-Fur. AMOX added dissolved in DMSO (AMOX conc.: 0.5 g/mL). **AMOX**: amoxicillin; **Di-Mal** (monomer): 1,8-dimaleimide-3,6-dioxaoctane; **GG**: guar gum; **IPN**: interpenetrating polymer network; **Suc**: porogen sucrose; **Di-Fur** (monomer): *N*,*N*’-difurfuryl-L-tartaramide; **Tri-Fur** (crosslinker): 2,3,5-tri-*O*-{[(furan-2-ylmethyl)carbamoyl]}-D-ribono-1,4-lactone.

## Data Availability

The data have been deposited in the idUS repository (Universidad de Sevilla, España) and can be accessed via the permanent link: https://hdl.handle.net/11441/175475 (accessed on 25 July 2025).

## Supplementary Materials

The following supporting information can be downloaded at: https://www.mdpi.com/article/10.3390/gels11100785/s1, Table S1: Quantitative analysis from digital microstructure studies blanks and IPN using FIJI, Table S2: Mathematical modeling and release kinetics of amoxicillin for the pre-loaded formulations (F1–F14) at pH 1.2; Table S3: Mathematical modeling and release kinetics of amoxicillin for the pre-loaded formulations (F1–F14) at pH 5.0; Table S4: Mathematical modeling and release kinetics of amoxicillin for the pre-loaded formulations (F1–F14) at pH 5.0; Table S5: Mathematical modeling and release kinetics of amoxicillin for the post-loaded formulations (B1.2 to B13.14 + AMOX) at pH 5.0; Table S6: AMOX release from single-step prepared and loaded formulations using sucrose as porogen, pH 1.2; Table S7: AMOX release from single-step prepared and loaded formulations using sucrose as porogen, pH 5.0; Table S8: AMOX release from single-step prepared and loaded formulations using PEG as porogen, pH 1.2; Table S9: AMOX release from single-step prepared and loaded formulations using PEG as porogen, pH 5.0; Table S10: AMOX release from post-loaded formulations using sucrose as porogen, pH 1.2; Table S11: AMOX release from post-loaded formulations using sucrose as porogen, pH 5.0; Table S12: AMOX release from post-loaded formulations using PEG as porogen, pH 1.2; Table S13: AMOX release from post-loaded formulations using PEG as porogen, pH 5.0.

## Abbreviations

The following abbreviations are used in this manuscript:

AMOX	Amoxicillin
API	Active pharmaceutical ingredient
CTS	Chitosan
DA	Diels–Alder
Di-Fur	*N*,*N*’-difurfuryl-L-tartaramide
Di-Mal	1,8-dimaleimide-3,6-dioxaoctane
DL	Drug loading
DMSO	Dimethyl sulfoxide
FLT	Floating lag time
FT	Floating time
FTIR	Fourier-transform infrared spectrum
GG	Guar gum
GRDDS	Gastro-retentive drug delivery systems
*H. pylori*	*Helicobacter pylori*
Hz	Hertz
IPN	Interpenetrating polymer network
Me4Si	Tetramethylsilane
MIC	Minimal inhibitory concentration
NMR	Nuclear magnetic resonance spectrum
PAA	Polyacrylic acid
PEG	Porogen polyethylene glycol
SEM	Scanning electron microscopy
SGF	Simulated gastric fluid
SI	Swelling index
Suc	Porogen sucrose
t_50_	Time (h) to 50% degradation of formulation mass
THF	Tetrahydrofuran
Tri-Fur	2,3,5-tri-*O*-{[(furan-2-ylmethyl)carbamoyl]}-D-ribono-1,4-lactone

## Appendix A. Formulation Codes for IPN Systems

The following table presents the codes for the different IPN systems prepared and described in this study. These codes are employed throughout the text to facilitate their identification and the understanding of the study.

**KEY CODE**	**COMPOSITION**
B1.2	GG-DA
B3.4	GG-DA-PEG_5_
B5.6	GG-DA-PEG_10_
B7.8	GG-DA-PEG_50_
B9.10	GG-DA-Suc_5_
B11.12	GG-DA-Suc_10_
B13.14	GG-DA-Suc_50_
F1	GG-DA-AMOX_25_
F2	GG-DA-AMOX_40_
F3	GG-DA-PEG_5_-AMOX_25_
F4	GG-DA-PEG_5_-AMOX_40_
F5	GG-DA-PEG_10_-AMOX_25_
F6	GG-DA-PEG_10_-AMOX_40_
F7	GG-DA-PEG_50_-AMOX_25_
F8	GG-DA-PEG_50_-AMOX_40_
F9	GG-DA-Suc_5_-AMOX_25_
F10	GG-DA-Suc_5_-AMOX_40_
F11	GG-DA-Suc_10_-AMOX_25_
F12	GG-DA-Suc_10_-AMOX_40_
F13	GG-DA-Suc_50_-AMOX_25_
F14	GG-DA-Suc_50_-AMOX_40_

**GG**: guar gum; **DA**: Diels–Alder polymer; **PEG**: polyethylene glycol-350; **Suc**: sucrose; **AMOX**: amoxicillin. The numbers indicate the concentration or ratio used, as described in Section 4.
