# Supplementary material for: Engineering Robust, Porous Guar Gum Hydrogels by One-Step Mild Synthesis: Impact of Porogen Choice on Rheology and Sustained Gastroretentive Amoxicillin Delivery
Source: Gels. 2025 Oct 1;11(10):785. doi: 10.3390/gels11100785 (PMC12564742; doi:10.3390/gels11100785)
Supplement: Supplementary file 1 [file gels-11-00785-s001.zip › gels-3813052-supplementary.pdf]

# Supplementary Materials

# Engineering Robust, Porous Guar Gum Hydrogels by One-Step Mild Synthesis: Impact of Porogen Choice on Rheology and Sustained Gastroretentive Amoxicillin Delivery

Fátima Díaz-Carrasco, M. Violante de-Paz *, Matea Katavić, Estefanía García-Pulido, Álvaro Santos-Medina, Lucía Muíña-Ramil, M. Gracia García-Martín and Elena Benito.

Departamento de Química Orgánica y Farmacéutica, Universidad de Sevilla, C/ Prof. García González, n.º 2, 41012-Sevilla, España.

* **Correspondence**: vdepaz@us.es

# Table of contents

1. **Microstructure of IPN and formulations** ........ 3

**Table S1**. Quantitative analysis from digital microstructure studies blanks and IPN using FIJI. ........ 3

2. **Kinetic studies on AMOX release** ........ 4

**Table S2**. Mathematical modeling and release kinetics of amoxicillin for the preloaded formulations (**F1-F14**) at pH 1.2 ........ 4

**Table S3**. Mathematical modeling and release kinetics of amoxicillin for the preloaded formulations (**F1-F14**) at pH 5.0 ........ 5

**Table S4**. Mathematical modeling and release kinetics of amoxicillin for the post-loaded formulations (**B1.2 to B13.14 + AMOX**) at pH 1.2. ........ 6

**Table S5**. Mathematical modeling and release kinetics of amoxicillin for the post-loaded formulations (**B1.2 to B13.14 + AMOX**) at pH 5.0. ........ 7

3. **Controlled drug release studies** ........ 8

**Table S6.** AMOX release from single-step prepared and loaded formulations using sucrose as porogen, pH 1.2. ........ 8

**Table S7.** AMOX release from single-step prepared and loaded formulations using sucrose as porogen, pH 5.0. ........ 8

**Table S8.** AMOX release from single-step prepared and loaded formulations using PEG as porogen, pH 1.2. ........ 9

**Table S9.** AMOX release from single-step prepared and loaded formulations using PEG as porogen, pH 5.0. ........ 9

**Table S10.** AMOX release from post-loaded formulations using sucrose as porogen, pH 1.2. ........ 10

**Table S11.** AMOX release from post-loaded formulations using sucrose as porogen, pH 5.0. ........ 10

**Table S12.** AMOX release from post-loaded formulations using PEG as porogen, pH 1.2. ........ 11

**Table S13.** AMOX release from post-loaded formulations using PEG as porogen, pH 5.0. ........ 11

## 1. Microstructure of IPN and formulations

**Table S1**. Quantitative analysis from digital microstructure studies blanks and IPN using FIJI.

| **Key code** | **B5.6** | **B7.8** | **B11.12** | **B13.14** | | **F14** |
|---|---|---|---|---|---|---|
| **Composition** | GG-DA-PEG$_{10}$ | GG-DA-PEG$_{50}$ | GG-DA-Suc$_{10}$ | GG-DA-Suc$_{50}$ | | GG-DA-Suc$_{50}$-AMOX$_{40}$ |
| **SEM magnitude** | 500X | 500X | 500X | 500X | 1,000X | 1,000X |
| **Perimeter (μm)** | 4.074 | 26.472 | 123.656 | 44.515 | 57.292 | 6.260 |
| **Diameter (μm)** | 1.297 | 8.426 | 39.361 | 14.169 | 18.237 | 1.992 |
| **Porous area (%)** | 6.415 | 13.200 | - | 7.456 | 9.330 | - |

## 2. Kinetic studies on AMOX release

**Table S2**. Mathematical modeling and release kinetics of amoxicillin for the preloaded formulations (**F1-F14**) at pH 1.2.

| **pH 1.2** | **Kinetic Parameters** | **Without porogen** | | **Porogen: PEG** | | | | | | **Porogen: Sucrose** | | | | | |
|---|---|---|---|---|---|---|---|---|---|---|---|---|---|---|---|
| **Porogen (%) →** | | **--** | **--** | **$PEG_5$** | | **$PEG_{10}$** | | **$PEG_{50}$** | | **$Suc_5$** | | **$Suc_{10}$** | | **$Suc_{50}$** | |
| **AMOX (%) →** | | **$AMOX_{25}$** | **$AMOX_{40}$** | **$AMOX_{25}$** | **$AMOX_{40}$** | **$AMOX_{25}$** | **$AMOX_{40}$** | **$AMOX_{25}$** | **$AMOX_{40}$** | **$AMOX_{25}$** | **$AMOX_{40}$** | **$AMOX_{25}$** | **$AMOX_{40}$** | **$AMOX_{25}$** | **$AMOX_{40}$** |
| **Kinetic Model ↓** | | **F1** | **F2** | **F3** | **F4** | **F5** | **F6** | **F7** | **F8** | **F9** | **F10** | **F11** | **F12** | **F13** | **F14** |
| **Higuchi** | $R^2$ | 0.8914 | 0.9694 | 0.8770 | 0.8680 | 0.8920 | 0.8080 | 0.8340 | 0.8180 | 0.9962 | 0.9496 | 0.9694 | 0.8732 | 0.8895 | 0.9188 |
| | $k_H$ | 16.96 | 12.66 | 29.22 | 34.99 | 12.43 | 27.06 | 8.89 | 6.76 | 23.28 | 13.97 | 9.04 | 10.03 | 8.52 | 5.85 |
| **Korsmeyer-Peppas** | $R^2$ | 0.9950 | 0.9973 | 0.9234 | 0.9277 | 0.9336 | 0.9277 | 0.9122 | 0.9231 | 0.9950 | 0.9132 | 0.9657 | 0.9065 | 0.9704 | 0.9809 |
| | $n$ | 0.230 | 0.351 | 0.300 | 0.231 | 0.248 | 0.164 | 0.184 | 0.176 | 0.451 | 0.511 | 0.395 | 0.250 | 0.224 | 0.268 |
| | $k_{KP}$ | 30.95 | 17.61 | 46.44 | 64.64 | 21.89 | 57.94 | 18.12 | 14.14 | 25.35 | 13.40 | 11.62 | 17.75 | 15.66 | 9.79 |

$R^2$: regression coefficient; $k_H$: Higuchi constant; $k_{KP}$: Korsmeyer − Peppas constant; $n$: difusion coefficient.

**Table S3**. Mathematical modeling and release kinetics of amoxicillin for the preloaded formulations (**F1-F14**) at pH 5.0.

| **pH 5.0** | **Kinetic Parameters** | **Without porogen** | | **Porogen: PEG** | | | | | | **Porogen: Sucrose** | | | | | |
|---|---|---|---|---|---|---|---|---|---|---|---|---|---|---|---|
| **Porogen (%) →** | | **--** | **--** | **$PEG_5$** | | **$PEG_{10}$** | | **$PEG_{50}$** | | **$Suc_5$** | | **$Suc_{10}$** | | **$Suc_{50}$** | |
| **AMOX (%) →** | | **$AMOX_{25}$** | **$AMOX_{40}$** | **$AMOX_{25}$** | **$AMOX_{40}$** | **$AMOX_{25}$** | **$AMOX_{40}$** | **$AMOX_{25}$** | **$AMOX_{40}$** | **$AMOX_{25}$** | **$AMOX_{40}$** | **$AMOX_{25}$** | **$AMOX_{40}$** | **$AMOX_{25}$** | **$AMOX_{40}$** |
| **Kinetic Model ↓** | | **F1** | **F2** | **F3** | **F4** | **F5** | **F6** | **F7** | **F8** | **F9** | **F10** | **F11** | **F12** | **F13** | **F14** |
| **Higuchi** | $R^2$ | 0.9663 | 0.9737 | 0.8741 | 0.8278 | 0.8843 | 0.9725 | 0.9104 | 0.8867 | 0.9579 | 0.8744 | 0.9480 | 0.8819 | 0.8763 | 0.8247 |
| | $k_H$ | 11.61 | 14.66 | 30.92 | 31.28 | 10.53 | 22.60 | 8.49 | 6.34 | 27.73 | 7.54 | 22.20 | 11.04 | 20.29 | 7.96 |
| **Korsmeyer-Peppas** | $R^2$ | 0.9909 | 0.9957 | 0.9136 | 0.9096 | 0.9855 | 0.9788 | 0.9000 | 0.9872 | 0.9381 | 0.9697 | 0.9522 | 0.7614 | 0.7405 | 0.8256 |
| | $n$ | 0.357 | 0.363 | 0.359 | 0.187 | 0.224 | 0.400 | 0.306 | 0.217 | 0.517 | 0.222 | 0.367 | 0.431 | 0.499 | 0.200 |
| | $k_{KP}$ | 16.04 | 19.82 | 42.11 | 63.68 | 19.47 | 28.66 | 13.37 | 11.83 | 28.06 | 14.09 | 30.48 | 13.90 | 22.50 | 15.85 |

$R^2$: regression coefficient; $k_H$: Higuchi constant; $k_{KP}$: Korsmeyer − Peppas constant; $n$: difusion coefficient.

**Table S4**. Mathematical modeling and release kinetics of amoxicillin for the post-loaded formulations (**B1.2 to B13.14 + AMOX**) at pH 1.2.

| **pH 1.2** | **Kinetic Parameters** | **Without porogen** | | **Porogen: PEG** | | | | | | **Porogen: Sucrose** | | | | | |
|---|---|---|---|---|---|---|---|---|---|---|---|---|---|---|---|
| **Porogen (%) →** | | **--** | **--** | **$PEG_5$** | | **$PEG_{10}$** | | **$PEG_{50}$** | | **$Suc_5$** | | **$Suc_{10}$** | | **$Suc_{50}$** | |
| **AMOX (%) →** | | **$AMOX_{25}$** | **$AMOX_{40}$** | **$AMOX_{25}$** | **$AMOX_{40}$** | **$AMOX_{25}$** | **$AMOX_{40}$** | **$AMOX_{25}$** | **$AMOX_{40}$** | **$AMOX_{25}$** | **$AMOX_{40}$** | **$AMOX_{25}$** | **$AMOX_{40}$** | **$AMOX_{25}$** | **$AMOX_{40}$** |
| **Kinetic Model ↓** | | **B1.2-$AMOX_{25}$** | **B1.2-$AMOX_{40}$** | **B3.4-$AMOX_{25}$** | **B3.4-$AMOX_{40}$** | **B5.6-$AMOX_{25}$** | **B5.6-$AMOX_{40}$** | **B7.8-$AMOX_{25}$** | **B7.8-$AMOX_{40}$** | **B9.10-$AMOX_{25}$** | **B9.10-$AMOX_{40}$** | **B11.12-$AMOX_{25}$** | **B11.12-$AMOX_{40}$** | **B13.14-$AMOX_{25}$** | **B13.14-$AMOX_{40}$** |
| **Higuchi** | $R^2$ | 0.9493 | 0.8576 | 0.9315 | 0.7394 | 0.9596 | 0.8228 | 0.9184 | 0.8410 | 0.9037 | 0.8462 | 0.7418 | 0.8612 | 0.7423 | 0.7866 |
| | $k_H$ | 34.82 | 21.71 | 27.01 | 20.37 | 35.25 | 26.75 | 29.78 | 26.27 | 35.37 | 27.22 | 25.24 | 18.43 | 27.99 | 24.69 |
| **Korsmeyer-Peppas** | $R^2$ | 0.9987 | 0.9825 | 0.9085 | 0.7721 | 0.9268 | 0.6809 | 0.8122 | 0.7162 | 0.9565 | 0.9759 | 0.5993 | 0.8040 | 0.7305 | 0.8606 |
| | $n$ | 0.306 | 0.187 | 0.385 | 0.104 | 0.790 | 1.611 | 0.950 | 0.909 | 0.270 | 0.182 | 0.733 | 0.870 | 0.118 | 0.152 |
| | $k_{KP}$ | 53.39 | 43.39 | 36.23 | 49.82 | 21.55 | 4.58 | 14.21 | 14.24 | 59.70 | 55.26 | 19.42 | 10.24 | 66.70 | 54.44 |

$R^2$: regression coefficient; $k_H$: Higuchi constant; $k_{KP}$: Korsmeyer − Peppas constant; $n$: difusion coefficient.

**Table S5**. Mathematical modeling and release kinetics of amoxicillin for the post-loaded formulations (**B1.2 to B13.14 + AMOX**) at pH 5.0.

| pH 5.0 | Kinetic Parameters | Without porogen | | Porogen: PEG | | | | | | Porogen: Sucrose | | | | | |
|---|---|---|---|---|---|---|---|---|---|---|---|---|---|---|---|
| Porogen (%) → | | -- | -- | $PEG_5$ | | $PEG_{10}$ | | $PEG_{50}$ | | $Suc_5$ | | $Suc_{10}$ | | $Suc_{50}$ | |
| AMOX (%) → | | $AMOX_{25}$ | $AMOX_{40}$ | $AMOX_{25}$ | $AMOX_{40}$ | $AMOX_{25}$ | $AMOX_{40}$ | $AMOX_{25}$ | $AMOX_{40}$ | $AMOX_{25}$ | $AMOX_{40}$ | $AMOX_{25}$ | $AMOX_{40}$ | $AMOX_{25}$ | $AMOX_{40}$ |
| Kinetic Model ↓ | | B1.2-$AMOX_{25}$ | B1.2-$AMOX_{40}$ | B3.4-$AMOX_{25}$ | B3.4-$AMOX_{40}$ | B5.6-$AMOX_{25}$ | B5.6-$AMOX_{40}$ | B7.8-$AMOX_{25}$ | B7.8-$AMOX_{40}$ | B9.10-$AMOX_{25}$ | B9.10-$AMOX_{40}$ | B11.12-$AMOX_{25}$ | B11.12-$AMOX_{40}$ | B13.14-$AMOX_{25}$ | B13.14-$AMOX_{40}$ |
| Higuchi | $R^2$ | 0.9321 | 0.9294 | 0.8969 | 0.9726 | 0.9190 | 0.9388 | 0.8762 | 0.8828 | 0.8805 | 0.8070 | 0.8751 | 0.8992 | 0.8227 | 0.8097 |
| | $k_H$ | 26.58 | 20.39 | 26.23 | 24.00 | 27.94 | 26.98 | 28.94 | 21.02 | 35.48 | 25.19 | 25.58 | 17.23 | 29.57 | 29.51 |
| Korsmeyer-Peppas | $R^2$ | 0.9868 | 0.9831 | 0.8805 | 0.9326 | 0.8663 | 0.8908 | 0.8478 | 0.7932 | 0.9323 | 0.9094 | 0.8242 | 0.8065 | 0.8648 | 0.6776 |
| | $n$ | 0.294 | 0.269 | 0.318 | 0.490 | 0.894 | 0.924 | 2.057 | 1.773 | 0.249 | 0.169 | 0.798 | 0.527 | 0.199 | 1.006 |
| | $k_{KP}$ | 42.26 | 33.71 | 40.61 | 25.63 | 14.38 | 12.55 | 1.92 | 6.21 | 63.08 | 53.52 | 15.98 | 17.95 | 59.14 | 13.83 |

$R^2$: regression coefficient; $k_H$: Higuchi constant; $k_{KP}$: Korsmeyer − Peppas constant; $n$: difusion coefficient.

## 3. Controlled drug release studies

**Table S6.** AMOX release from single-step prepared and loaded formulations using sucrose as porogen, pH 1.2.

| | $Suc_5$-$AMOX_{25}$ | | $Suc_5$-$AMOX_{40}$ | | $Suc_{10}$-$AMOX_{25}$ | | $Suc_{10}$-$AMOX_{40}$ | | $Suc_{50}$-$AMOX_{25}$ | | $Suc_{50}$-$AMOX_{40}$ | |
|---|---|---|---|---|---|---|---|---|---|---|---|---|
| **Time (h)** | AMOX (mg) | AMOX (mg/L)[a] | AMOX (mg) | AMOX (mg/L)[a] | AMOX (mg) | AMOX (mg/L)[a] | AMOX (mg) | AMOX (mg/L)[a] | AMOX (mg) | AMOX (mg/L)[a] | AMOX (mg) | AMOX (mg/L)[a] |
| **0.00** | 0.00 | 0.00 | 0.00 | 0.00 | 0.00 | 0.00 | 0.00 | 0.00 | 0.00 | 0.00 | 0.00 | 0.00 |
| **1.00** | 8.72 | 5.13 | 9.58 | 5.64 | 3.61 | 2.12 | 11.44 | 6.73 | 5.31 | 3.13 | 6.41 | 3.77 |
| **2.00** | 2.82 | 1.65 | 2.36 | 1.39 | 1.91 | 1.13 | 2.31 | 1.36 | 0.47 | 0.28 | 1.43 | 0.84 |
| **3.00** | 2.08 | 1.22 | 1.55 | 0.91 | 0.62 | 0.34 | -10.34 | -6.08 | 1.06 | 0.62 | 1.37 | 0.81 |
| **5.00** | 4.68 | 2.75 | 10.79 | 6.35 | 1.19 | 0.70 | 15.37 | 9.04 | 0.75 | 0.44 | 0.56 | 0.33 |
| **8.00** | 3.52 | 2.07 | 0.55 | 0.32 | 1.08 | 0.64 | -0.40 | -0.24 | 0.65 | 0.38 | 1.55 | 0.91 |

[a]Calculation AMOX concentration. with consideration given to the average volume of a full stomach (1.7 L)

**Table S7.** AMOX release from single-step prepared and loaded formulations using sucrose as porogen, pH 5.0.

| | $Suc_5$-$AMOX_{25}$ | | $Suc_5$-$AMOX_{40}$ | | $Suc_{10}$-$AMOX_{25}$ | | $Suc_{10}$-$AMOX_{40}$ | | $Suc_{50}$-$AMOX_{25}$ | | $Suc_{50}$-$AMOX_{40}$ | |
|---|---|---|---|---|---|---|---|---|---|---|---|---|
| **Time (h)** | AMOX (mg) | AMOX (mg/L)[a] | AMOX (mg) | AMOX (mg/L)[a] | AMOX (mg) | AMOX (mg/L)[a] | AMOX (mg) | AMOX (mg/L)[a] | AMOX (mg) | AMOX (mg/L)[a] | AMOX (mg) | AMOX (mg/L)[a] |
| **0.00** | 0.00 | 0.00 | 0.00 | 0.00 | 0.00 | 0.00 | 0.00 | 0.00 | 0.00 | 0.00 | 0.00 | 0.00 |
| **1.00** | 4.75 | 2.79 | 2.04 | 1.20 | 3.86 | 2.27 | 8.72 | 5.12 | 8.65 | 5.09 | 3.74 | 2.20 |
| **2.00** | 5.17 | 3.04 | 1.40 | 0.82 | 3.21 | 1.89 | 0.71 | 0.41 | 1.17 | 0.69 | 0.30 | 0.18 |
| **3.00** | 4.93 | 2.90 | 0.73 | 0.43 | 2.09 | 1.23 | 1.50 | 0.8 | 0.41 | 0.24 | 0.32 | 0.19 |
| **5.00** | 0.48 | 0.28 | 1.31 | 0.77 | 1.61 | 0.95 | 1.39 | 0.89 | 2.17 | 1.28 | 1.42 | 0.83 |
| **8.00** | 8.65 | 5.09 | 9.10 | 5.35 | 9.54 | 5.61 | 7.39 | 4.34 | 5.69 | 3.35 | 9.77 | 5.72 |

[a] Calculation AMOX concentration. with consideration given to the average volume of a full stomach (1.7 L)

**Table S8.** AMOX release from single-step prepared and loaded formulations using PEG as porogen, pH 1.2.

| | $PEG_5$-$AMOX_{25}$ | | $PEG_5$-$AMOX_{40}$ | | $PEG_{10}$-$AMOX_{25}$ | | $PEG_{10}$-$AMOX_{40}$ | | $PEG_{50}$-$AMOX_{25}$ | | $PEG_{50}$-$AMOX_{40}$ | |
|---|---|---|---|---|---|---|---|---|---|---|---|---|
| **Time (h)** | AMOX (mg) | AMOX (mg/L)[a] | AMOX (mg) | AMOX (mg/L)[a] | AMOX (mg) | AMOX (mg/L)[a] | AMOX (mg) | AMOX (mg/L)[a] | AMOX (mg) | AMOX (mg/L)[a] | AMOX (mg) | AMOX (mg/L)[a] |
| **0.00** | 0.00 | 0.00 | 0.00 | 0.00 | 0.00 | 0.00 | 0.00 | 0.00 | 0.00 | 0.00 | 0.00 | 0.00 |
| **1.00** | 14.74 | 8.67 | 40.55 | 23.85 | 6.92 | 4.07 | 37.11 | 21.83 | 5.77 | 3.40 | 8.98 | 5.28 |
| **2.00** | 3.31 | 1.95 | 13.38 | 7.87 | 2.33 | 1.37 | 8.11 | 4.77 | 1.61 | 0.95 | 2.17 | 1.28 |
| **3.00** | 8.20 | 4.82 | 3.32 | 1.95 | 0.72 | 0.43 | 1.45 | 0.85 | 0.02 | 0.01 | 0.58 | 0.34 |
| **5.00** | -2.41 | -1.42 | 5.65 | 3.33 | 0.35 | 0.21 | 4.75 | 2.79 | 0.46 | 0.27 | 0.86 | 0.51 |
| **8.00** | 3.60 | 2.12 | 3.70 | 2.18 | 1.85 | 1.09 | 0.93 | 0.55 | 0.95 | 0.56 | 0.48 | 0.28 |

[a] Calculation AMOX concentration. with consideration given to the average volume of a full stomach (1.7 L)

**Table S9.** AMOX release from single-step prepared and loaded formulations using PEG as porogen, pH 5.0.

| | $PEG_5$-$AMOX_{25}$ | | $PEG_5$-$AMOX_{40}$ | | $PEG_{10}$-$AMOX_{25}$ | | $PEG_{10}$-$AMOX_{40}$ | | $PEG_{50}$-$AMOX_{25}$ | | $PEG_{50}$-$AMOX_{40}$ | |
|---|---|---|---|---|---|---|---|---|---|---|---|---|
| **Time (h)** | AMOX (mg) | AMOX (mg/L)[a] | AMOX (mg) | AMOX (mg/L)[a] | AMOX (mg) | AMOX (mg/L)[a] | AMOX (mg) | AMOX (mg/L)[a] | AMOX (mg) | AMOX (mg/L)[a] | AMOX (mg) | AMOX (mg/L)[a] |
| **0.00** | 0.00 | 0.00 | 0.00 | 0.00 | 0.00 | 0.00 | 0.00 | 0.00 | 0.00 | 0.00 | 0.00 | 0.00 |
| **1.00** | 14.26 | 8.39 | 40.32 | 23.72 | 6.43 | 3.78 | 18.29 | 10.76 | 4.05 | 2.38 | 7.99 | 4.70 |
| **2.00** | 2.84 | 1.67 | 11.10 | 6.53 | 1.05 | 0.62 | 7.25 | 4.26 | 2.09 | 1.23 | 0.87 | 0.51 |
| **3.00** | 2.21 | 1.30 | 1.02 | 0.60 | 1.15 | 0.67 | 6.01 | 3.54 | 0.44 | 0.26 | 1.31 | 0.77 |
| **5.00** | 12.18 | 7.17 | 6.33 | 3.73 | 0.67 | 0.39 | 5.46 | 3.21 | 0.51 | 0.30 | 0.98 | 0.58 |
| **8.00** | -5.30 | -3.12 | 1.17 | 0.69 | 0.91 | 0.53 | 4.64 | 2.73 | 0.94 | 0.55 | 1.26 | 0.74 |

[a] Calculation AMOX concentration. with consideration given to the average volume of a full stomach (1.7 L)

**Table S10.** AMOX release from post-loaded formulations using sucrose as porogen, pH 1.2.

| | **B9.10-AMOX25** | | **B9.10-AMOX40** | | **B11.12-AMOX25** | | **B11.12-AMOX40** | | **B13.14-AMOX25** | | **B13.14-AMOX40** | |
|---|---|---|---|---|---|---|---|---|---|---|---|---|
| **Time (h)** | AMOX (mg) | AMOX (mg/L)[a] | AMOX (mg) | AMOX (mg/L)[a] | AMOX (mg) | AMOX (mg/L)[a] | AMOX (mg) | AMOX (mg/L)[a] | AMOX (mg) | AMOX (mg/L)[a] | AMOX (mg) | AMOX (mg/L)[a] |
| **0.00** | 0.00 | 0.00 | 0.00 | 0.00 | 0.00 | 0.00 | 0.00 | 0.00 | 0.00 | 0.00 | 0.00 | 0.00 |
| **1.00** | 18.94 | 11.14 | 37.18 | 21.87 | 3.69 | 2.17 | 4.77 | 2.80 | 21.86 | 12.86 | 35.28 | 20.76 |
| **2.00** | 5.76 | 3.39 | 3.17 | 1.86 | 16.40 | 9.65 | 10.64 | 6.26 | 1.20 | 0.71 | 4.22 | 2.48 |
| **3.00** | 6.05 | 3.56 | 5.35 | 3.15 | -0.06 | -0.04 | 11.89 | 7.00 | 4.91 | 2.89 | 7.04 | 4.14 |
| **5.00** | 0.25 | 0.14 | 4.65 | 2.73 | 0.13 | 0.08 | 1.84 | 1.09 | -1.41 | -0.83 | 0.14 | 0.08 |
| **8.00** | 2.30 | 1.35 | 2.70 | 1.59 | 0.84 | 0.50 | 0.45 | 0.26 | 1.00 | 0.59 | 1.03 | 0.60 |

[a] Calculation AMOX concentration. with consideration given to the average volume of a full stomach (1.7 L)

**Table S11.** AMOX release from post-loaded formulations using sucrose as porogen, pH 5.0.

| | **B9.10-AMOX25** | | **B9.10-AMOX40** | | **B11.12-AMOX25** | | **B11.12-AMOX40** | | **B13.14-AMOX25** | | **B13.14-AMOX40** | |
|---|---|---|---|---|---|---|---|---|---|---|---|---|
| **Time (h)** | AMOX (mg) | AMOX (mg/L)[a] | AMOX (mg) | AMOX (mg/L)[a] | AMOX (mg) | AMOX (mg/L)[a] | AMOX (mg) | AMOX (mg/L)[a] | AMOX (mg) | AMOX (mg/L)[a] | AMOX (mg) | AMOX (mg/L)[a] |
| **0.00** | 0.00 | 0.00 | 0.00 | 0.00 | 0.00 | 0.00 | 0.00 | 0.00 | 0.00 | 0.00 | 0.00 | 0.00 |
| **1.00** | 19.79 | 11.64 | 34.27 | 20.16 | 3.96 | 2.33 | 9.48 | 5.58 | 18.46 | 10.86 | 4.83 | 2.84 |
| **2.00** | 6.50 | 3.83 | 6.43 | 3.78 | 6.78 | 3.99 | 11.29 | 6.64 | 5.06 | 2.98 | 30.96 | 18.21 |
| **3.00** | 2.55 | 1.50 | 4.34 | 2.55 | 8.02 | 4.71 | 5.66 | 3.33 | 2.70 | 1.60 | 6.83 | 4.02 |
| **5.00** | 3.12 | 1.84 | 2.97 | 1.75 | 1.83 | 1.08 | 2.18 | 1.28 | 1.62 | 0.95 | 4.83 | 2.84 |
| **8.00** | 1.37 | 0.80 | 0.13 | 0.07 | 0.06 | 0.04 | 0.83 | 0.49 | -0.08 | -0.05 | -0.44 | -0.26 |

[a] Calculation AMOX concentration. with consideration given to the average volume of a full stomach (1.7 L)

**Table S12.** AMOX release from post-loaded formulations using PEG as porogen, pH 1.2.

| | **B3.4-AMOX25** | | **B3.4-AMOX40** | | **B5.6-AMOX25** | | **B5.6-AMOX40** | | **B7.8-AMOX25** | | **B7.8-AMOX40** | |
|---|---|---|---|---|---|---|---|---|---|---|---|---|
| **Time (h)** | AMOX (mg) | AMOX (mg/L)[a] | AMOX (mg) | AMOX (mg/L)[a] | AMOX (mg) | AMOX (mg/L)[a] | AMOX (mg) | AMOX (mg/L)[a] | AMOX (mg) | AMOX (mg/L)[a] | AMOX (mg) | AMOX (mg/L)[a] |
| **0.00** | 0.00 | 0.00 | 0.00 | 0.00 | 0.00 | 0.00 | 0.00 | 0.00 | 0.00 | 0.00 | 0.00 | 0.00 |
| **1.00** | 10.89 | 6.41 | 32.53 | 19.13 | 5.90 | 3.47 | 1.09 | 0.64 | 3.10 | 1.82 | 5.59 | 3.29 |
| **2.00** | 5.96 | 3.51 | 5.51 | 3.24 | 8.86 | 5.21 | 24.96 | 14.68 | 10.80 | 6.35 | 23.98 | 14.11 |
| **3.00** | 3.57 | 2.10 | -2.94 | -1.73 | 4.41 | 2.59 | 11.02 | 6.48 | 4.38 | 2.57 | 8.90 | 5.23 |
| **5.00** | 2.88 | 1.69 | 4.90 | 2.88 | 7.95 | 4.68 | 3.09 | 1.82 | 3.61 | 2.13 | 2.77 | 1.63 |
| **8.00** | 0.83 | 0.49 | 0.99 | 0.58 | 3.21 | 1.89 | 2.29 | 1.35 | 3.32 | 1.95 | 1.38 | 0.81 |

[a] calculation AMOX concentration. with consideration given to the average volume of a full stomach (1.7 L)

**Table S13.** AMOX release from post-loaded formulations using PEG as porogen, pH 5.0.

| | **B3.4-AMOX25** | | **B3.4-AMOX40** | | **B5.6-AMOX25** | | **B5.6-AMOX40** | | **B7.8-AMOX25** | | **B7.8-AMOX40** | |
|---|---|---|---|---|---|---|---|---|---|---|---|---|
| **Time (h)** | AMOX (mg) | AMOX (mg/L)[a] | AMOX (mg) | AMOX (mg/L)[a] | AMOX (mg) | AMOX (mg/L)[a] | AMOX (mg) | AMOX (mg/L)[a] | AMOX (mg) | AMOX (mg/L)[a] | AMOX (mg) | AMOX (mg/L)[a] |
| **0.00** | 0.00 | 0.00 | 0.00 | 0.00 | 0.00 | 0.00 | 0.00 | 0.00 | 0.00 | 0.00 | 0.00 | 0.00 |
| **1.00** | 12.28 | 7.22 | 15.16 | 8.92 | 3.51 | 2.06 | 6.25 | 3.67 | 0.29 | 0.17 | 0.71 | 0.42 |
| **2.00** | 5.55 | 3.27 | 12.65 | 7.44 | 8.11 | 4.77 | 15.40 | 9.06 | 5.41 | 3.19 | 10.92 | 6.42 |
| **3.00** | 3.68 | 2.16 | 2.24 | 1.32 | 4.45 | 2.62 | 7.60 | 4.47 | 5.76 | 3.39 | 12.20 | 7.18 |
| **5.00** | 1.60 | 0.94 | 8.64 | 5.09 | 6.47 | 3.81 | 3.39 | 1.99 | 6.42 | 3.78 | 3.04 | 1.79 |
| **8.00** | 0.67 | 0.39 | 4.70 | 2.77 | 0.31 | 0.18 | 17.35 | 10.21 | 7.56 | 4.44 | 8.94 | 5.26 |

[a] Calculation AMOX concentration. with consideration given to the average volume of a full stomach (1.7 L)
